# Stool biomarkers as measures of enteric pathogen infection in infants from Addis Ababa informal settlements

**DOI:** 10.1371/journal.pntd.0011112

**Published:** 2023-02-21

**Authors:** Leon M. Espira, Gwenyth O. Lee, Kaleab Baye, Andrew D. Jones, Nancy G. Love, Joseph N. S. Eisenberg

**Affiliations:** 1 Department of Epidemiology, University of Michigan School of Public Health, Ann Arbor, Michigan, United States of America; 2 Rutgers Global Health Institute & Department of Biostatistics and Epidemiology School of Public Health Rutgers, The State University of New Jersey, New Brunswick, New Jersey, United States of America; 3 Center for Food Science and Nutrition, College of Natural and Computational Sciences, Addis Ababa University, Addis Ababa, Ethiopia; 4 Department of Nutritional Sciences, University of Michigan School of Public Health, Ann Arbor, Michigan, United States of America; 5 Department of Civil and Environmental Engineering, University of Michigan, Ann Arbor, Michigan, United States of America; Washington University School of Medicine, UNITED STATES

## Abstract

Frequent enteric infections in children may be an important cause of growth faltering; however, we do not fully understand the mechanisms by which pathogen infections and the physiological responses to these infections result in poorer growth. Commonly used protein fecal biomarkers (anti-alpha trypsin, neopterin, and myeloperoxidase) provide broad immunological information on an inflammatory response; however, they do not provide information on non-immune processes (e.g., gut integrity) that may be important indicators of chronic end states such as environmental enteric dysfunction (EED). To explore how additional biomarkers will better inform which physiological pathways (both immune and non-immune) are impacted by pathogen exposure we added to the traditional panel of 3 protein fecal biomarkers 4 novel fecal mRNA transcript biomarkers (sucrase isomaltase, caudal homeobox 1, S100A8, and mucin 12) and analyzed stool samples from infants living in informal settlements in Addis Ababa, Ethiopia. To test how this expanded biomarker panel captures distinct pathogen exposure processes we used two different scoring systems. First, we used a theory-based approach to assign each biomarker to specific physiological attributes based on prior understanding of each biomarker. Second, we used data reduction methods to categorize biomarkers and then assign physiological attributes to those categories. We used linear models to examine the association between the derived biomarker scores (based on mRNA and protein levels) and stool pathogen gene counts to determine pathogen specific effects on gut physiology and immune responses. Inflammation scores were positively associated with *Shigella* and enteropathogenic *E*.*Coli* (EPEC) infection, while gut integrity scores were negatively associated with *Shigella*, EPEC and, shigatoxigenic *E*.*coli* (STEC) infection. Our expanded panel of biomarkers hold promise as tools to measure systemic outcomes of enteric pathogen infection. mRNA biomarkers complement established protein biomarkers by providing important cell-specific physiological and immunological consequences of pathogen carriage that can lead to chronic end states such as EED.

## Author summary

Frequent enteric infections in children may be an important cause of growth faltering. Mechanisms by which pathogen infections result in poorer growth, however, are not fully understood. Commonly used biomarkers provide information on broad immunological processes but not on non-immune processes (e.g., gut integrity) that may be important indicators of overall health. In this manuscript we explore how additional biomarkers can expand our understanding on which immune and non-immune pathways are impacted by pathogen exposure. To do this we developed a panel consisting of 3 commonly used and 4 novel biomarkers. We analyzed stool samples from infants living in informal settlements in Addis Ababa, Ethiopia. To test how this expanded biomarker panel captures distinct pathological processes we used two scoring systems and examined their association with stool pathogen gene counts. We found that *Shigella* and enteropathogenic *E*. *Coli* (EPEC) were positively associated in inflammation, while the integrity of intestinal tissue was negatively associated with *Shigella*, EPEC and shigatoxigenic *E*.*coli* (STEC) infection. Our biomarker panel holds promise as a tool to measure both immune and non-immune outcomes of pathogen infection.

## Introduction

Infant growth faltering remains a persistent concern in low- and middle-income countries globally, where approximately 165 million children under 5 years of age are stunted [1]. It has long been recognized that enteric infections are an important cause of growth faltering; however, the mechanisms by which frequent pathogen infections and the physiological responses to these infections result in poorer growth remain poorly understood. One putative mechanistic linking enteric pathogen infection and growth faltering is a condition known as environmental enteric dysfunction (EED).

EED results in altered small bowel physiology due to repeated enteric infections. EED has demonstrable histological features such as villous flattening, crypt hyperplasia and lymphocytic infiltration of the lamina propria coupled with chronic intestinal inflammation [2–6], which have been associated with long-term growth faltering and stunting. EED can compromise the integrity of the gut and allow potentially pathogenic bacteria to cross the gut wall, triggering a chronic inflammatory process [7]. Inflammation may directly down-regulate growth, as well as indirectly affect growth through appetite suppression [7,8]. Because EED may compromise the integrity of the intestinal wall, an abnormally large number of white blood cells can infiltrate the gut, forcing an infant’s metabolism to manage a chronically stressed immune system [7,9,10].

Diagnosing EED has been a challenge, potentially because EED is likely not a single syndrome, but the result of multiple contributing processes that may differ geographically [11]. An effective diagnostic tool, therefore, is one that captures specific, critical aspects of gut health, such as enterocyte damage. Asymptomatic pathogen carriage is common among young children living without access to improved water and sanitation. This potentially hinders the use of diagnostic techniques that rely on the measurement of immunological responses, since asymptomatic pathogen carriage often results in transient immune activation [12,13]. Asymptomatic pathogen carriage still causes cellular and tissue damage, reinforcing the need for diagnostic tools that provide cell and tissue specific measures of immune state and tissue integrity.

The gold standard for diagnosis of EED is the histological examination of intestinal tissue. However, because of the invasive nature of collecting biopsies from very young children, there have been concerted efforts to develop alternative non-invasive tests. The most common noninvasive test is the lactulose: mannitol (L:M) urine test. The L:M test is designed to measure intestinal permeability but provides no information on the immunological state of subjects [9,10]. The L:M test is also challenging to administer and results are hard to compare across dosing and urine collection protocols and analytic platforms [14].

Another frequently reported EED measure is a biomarker panel consisting of three proteins isolated from stool: alpha-1-antitrypsin (AAT), neopterin, and myeloperoxidase (MPO) [15]. Human alpha-1 antitrypsin is a water soluble glycoprotein [16]. During intestinal inflammation, AAT is extraverted into the gut and is therefore a marker of intestinal permeability and protein loss [15]. Neopterin is synthesized primarily by activated monocytes, macrophages, dendritic cells, and endothelial cells [17]. The production of neopterin by macrophages is a marker of INF-γ mediated activation of macrophages by TH1 cells, indicative of an adaptive immune response; i.e., the pathogens driving this response have previously been encountered [18,19]. Neopterin has been used to diagnose autoimmune diseases such as celiac disease, which is considered a clinical and histopathological analog of EED [15]. MPO is a marker of the neutrophil response and has been correlated with disease activity in inflammatory bowel disease [15].

These three protein stool biomarkers are easier to measure than the lactulose: mannitol ratio and provide greater immunological detail and information about permeability. To improve specificity even more, mRNA transcripts specific for intestinal inflammation have recently been investigated [20–23]. Several features make these transcripts promising for the assessment of EED: 1) they are able to target and measure a range of intestinal processes related to the immune system much like AAT, neopterin, and MPO; 2) they are able to measure other non-immune processes such as epithelial state and nutrient absorption that are important indicators of EED; and 3) their measurement can be multiplexed, allowing for multiple markers to be measured simultaneously. Because mRNA transcript biomarkers can be cell specific, they may be particularly valuable in providing cellular level information on specific physiological and immunological processes.

These mRNA transcripts have been described and measured in populations of rural infants and children in Malawi and Sierra Leone, where they showed promise as indicators of EED based on their association with L:M test results and inflammatory indicators [20–25]. However, several questions remain about the use of these biomarkers for the assessment of EED. Of particular interest is whether these biomarkers accurately measure the features of EED (i.e., gut integrity and immunological activation) that are critical to child health.

In this manuscript, we examine the degree to which our expanded biomarker panel can distinguish among pathogens; specifically, we aim to distinguish between those that are enteroinvasive compared to those that attach to enterocyte surfaces, and those that form biofilms. We do this by analyzing stool samples from infants living in informal settlements in Addis Ababa, Ethiopia.

Our biomarker panel comprises three established fecal protein biomarkers (AAT, neopterin, and MPO) and 4 novel mRNA transcript biomarkers (sucrase isomaltase (SI), caudal homeobox 1 (Cdx1), S100A8 and mucin 12). SI and Cdx1 were selected to inform non-inflammatory processes, those related to gut integrity. SI is located on the brush border membrane of enterocytes, and SI levels are reduced with mucosal injury [26]. Caudal homeobox proteins are global transcription factors, and a close homologue of Cdx1 has been shown to transactivate SI [27]. Given the similarities, we argue that Cdx1 can be used as a marker of gut integrity along with SI. S100A8, which is part of the heterodimeric protein S100A8/A9 (calprotectin) [28], was selected to mirror the neutrophil response measured by MPO. Finally, Mucin 12 is a membrane bound mucin constitutively expressed by enterocytes mainly in the colon, and is therefore a third potential marker of gut integrity [29,30].

## Materials and methods

### Ethical approval

Ethical approval for sample collection and surveys was granted by institutional review boards at the University of Michigan (HUM00115103), and the Addis Ababa University (IRB/029/2017). Parents or legal guardians gave verbal, informed consent prior to participation or collection of data. Fecal samples were transported with clearance obtained from the Food, Medicine, and Health Care Administration, and Control Authority in Ethiopia.

### Study population

Stool samples were collected in 2018 from 136 infants aged 6–23 months across 12 localities in Addis Ababa, Ethiopia. Based on available resources, our goal was to obtain 8–12 stool samples per locality (across all 12 localities enrolled in our larger water and sanitation study). Within each locality, health extension workers identified all households with infants aged 6–23 months and compiled them into a list. In the larger study, every second or third household was systematically selected (depending on the number of households in the locality). For this biomarker study, 12 households from each locality were randomly selected from the large study sample and asked to provide stool samples. A key informant was asked to respond to a survey on sanitation access and consent to the collection of anthropometric data.

### Stool sample processing

After collection, stool samples were catalogued and scored for consistency (formed, soft or watery). Samples were stored at 4°C overnight before processing the following morning. Aliquots of the stool samples, to be used for ELISA biomarker measurements, was stored at -80°C. Aliquots were also processed for nucleic acid extraction using the ZymoBiomics DNA/RNA Mini Kit (Zymo Research, Irvine, CA).

### ELISA protein biomarker measurements

ELISA’s for three protein biomarkers (AAT, neopterin, and MPO) were run on-site at the Ethiopian Institute of Public Health as per the manufacturer’s instructions. AAT levels were determined using the Human Alpha-1-Antitrypsin ELISA from Biovendor Research and Diagnostic Products (Brno, Czech Republic) kit. Neopterin levels were determined using the GenWay Biotech Inc. (San Diego, USA) Neopterin ELISA. MPO levels were measured using the IDK MPO ELISA from Immunodiagnostik AG (Bensheim, Germany). Prior to running the ELISA assays, stool aliquots were thawed on ice and thereafter stored at 4°C until the assays were run.

### Nucleic acid extraction

Standard ZymoBiomics DNA/RNA Mini Kit (Zymo Research, Irvine, CA) protocol for nucleic acid isolation from fecal samples was used. Briefly, 200mg of stool was weighed out and placed in screw cap microcentrifuge tubes containing the DNA/RNA Shield Lysis Buffer. The tubes were packed in leak proof containers for transport to the University of Michigan where DNA and RNA were extracted as specified in the manufacturer’s protocol within two months of sample collection. The quality and concentration of the extracted nucleic acids was measured using a Nanodrop Spectrophotometer (Thermo Scientific, Waltham, MA). The DNA and RNA were used to detect mRNA transcripts and pathogens.

### Droplet digital PCR detection of mRNA transcripts

Sample mRNA transcript counts were quantified using the QX200 Droplet Digital PCR (ddPCR) system (Bio-Rad, Hercules, CA) using duplexed FAM and VIC TaqMan assays. Assay setup and cycling conditions are provided in the S1 Text.

### Droplet digital PCR quantification of stool pathogen gene counts

Pathogen gene counts were quantified using the QX200 Droplet Digital PCR system (Bio-Rad, Hercules, CA). Assay setup and cycling conditions are provided in the S2 Text. Stool samples were screened for six bacteria (enteroaggregative *E*.*coli* (EAEC), enteropathogenic *E*.*coli* (EPEC), enterotoxigenic *E*.*coli* (ETEC), shigatoxigenic *E*.*coli* (STEC), *Shigella*, and *Campylobacter spp*), two protozoans (*Giardia lamblia*, and *Cryptosporidium spp*), and one virus (norovirus GI and GII). EPEC was screened for two gene targets (S1 Table).

### Droplet digital PCR data processing

All sample quantification was carried out using QuantaSoft software (Bio-Rad, Hercules, CA). Wells were checked and samples with <10,000 accepted droplets were rerun. To check the inter assay variability, 1/3 of the samples on a 96-well plate were randomly selected to be re-run for both mRNA transcript and pathogen quantification, and coefficients of variation (CV) were calculated. If more than 5% of re-run samples had CVs higher than 15%, the entire 96-well plate from which the samples were selected from was re-run.

The quantification of mRNA transcripts was carried out by visually setting thresholds to distinguish between negative and positive droplets [20,21]. The concentration in each well was then normalized to GAPDH and results presented as Target/GAPDH ratios.

To quantify stool pathogen gene counts, the threshold for differentiating negative from positive droplets was defined as one standard deviation above the negative droplet threshold on the no template controls [31,32]. Following the setting of the threshold, all wells were visually inspected. Wells with less than three positive droplets were considered negative and assigned a value equal to 0.1 prior to the calculation of final pathogen gene concentrations. This avoids the log transformation of zero values [31]. Final concentrations of gene copies per 200mg of stool were obtained by multiplying by appropriate dilution factors. Final concentrations were then converted to log_10_.

The data from the ELISA and ddPCR analysis is deposited in Dryad under the unique digital object identifier (DOI): doi:10.5061/dryad.7pvmcvdwq [33].

### Development of Physiological Scores and Statistical Analysis

**Fig 1:**
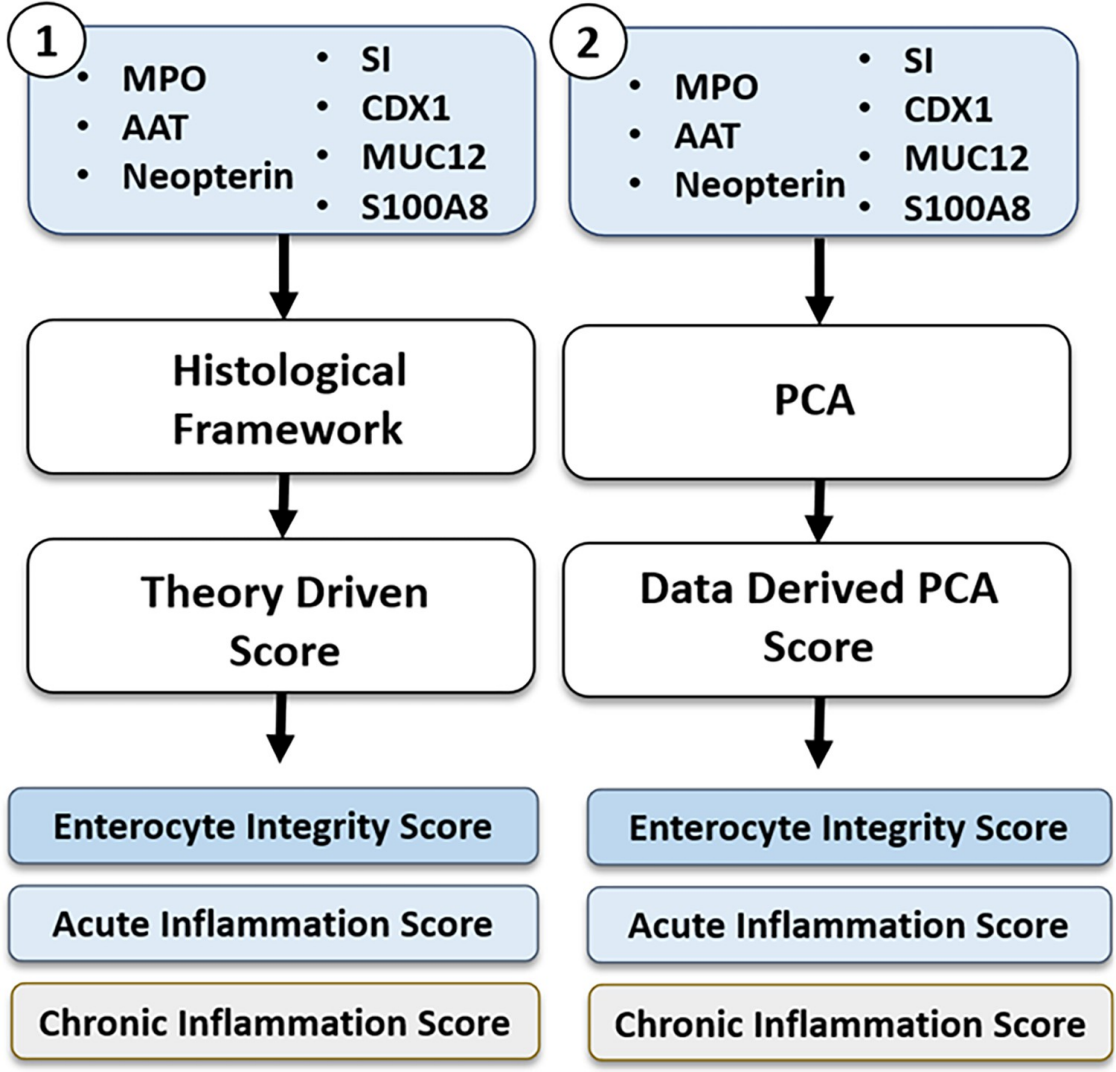
Derivation of the two scoring systems for the analysis. The protein biomarkers were anti-alpha trypsin (AAT), neopterin, myeloperoxidase (MPO). The mRNA biomarkers were sucrase isomaltase (SI), caudal homeobox 1 (CDX1), S100A8, and mucin (MUC12).

We develop two summary scores based on our panel of 3 protein and 4 mRNA biomarkers: 1) “theory driven” scores similar to that described by Liu *et al*. (2020) (S2 Table) [11] and 2) “data derived” PCA scores (Fig 1).

The “theory driven” scores were derived by sorting the biomarkers into quintiles with the lowest quintile assigned a grade score of 0 and the highest quintile assigned a grade score of 4 (see Eqs 1–4). Four score types were derived using different subsets of biomarkers: 1) a “Enterocyte Integrity Score” consisting of SI, Cdx1 and mucin 12; 2) an “Acute Inflammation Score” consisting of S100A8, MPO and AAT that all measured the neutrophil response; 3) a “Chronic Inflammation Score” where neopterin is a proxy for the adaptive immune response; and 4) an “Inflammation Score” consisting of S100A8, MPO, AAT and neopterin. Given the highly contaminated environments of these infants, the “Chronic Inflammation Score” can be treated as a measure of frequently encountered pathogens, which often trigger an immune response. We therefore use neopterin as a proxy for the adaptive immune response. In the compound scores (Enterocyte Integrity, Acute Inflammation, and Inflammation), each of the biomarkers contributed equally to the scores.


$$\begin{array}{c} Health\;Score=SI\;Quintile+CDX1\;Quintile+Mucin12\;Quintile\\ \left[0-4]\;[0-4]\;[0-4\right]\\ Maximum\;Score=12 \end{array}$$ (1)

$$\begin{array}{c} Acute\;Inflammation\;Score=S100A8\;Quintile+MPO\;Quintile+AAT\;Quintile\\ \left[0-4]\;[0-4]\;[0-4\right]\\ Maximum\;Score=12 \end{array}$$ (2)

$$\begin{array}{c} Chronic\;Inflammation\;Score=Neopterin\;Quintile\\ \left[0-4\right]\\ Maximum\;Score=4 \end{array}$$ (3)

$$\begin{array}{c} Inflammation\;Score=S100A8\;Quintile+MPO\;Quintile+AAT\;Quintile+Neopterin\;Quintile\\ \left[0-4]\;[0-4]\;[0-4][0-4\right]\\ Maximum\;Score=16 \end{array}$$ (4)

For the creation of the “data derived” score, we first removed the outliers using the interquartile range method (IQR) method where any observations that were more than 1.5 IQR below Q1 or more than 1.5 IQR above Q3 were considered outliers and removed [34]. In total 13 samples were removed from the data set. The remaining values were standardized around the mean. The PCA, was run using factor loading values of > 0.4 or < -0.4. Components were selected that explained at least 80% of the cumulative proportion of the variance based on the Kaiser-Guttman rule and the Scree test [35].

An infant’s score was arrived at by summing the product of the measured biomarker levels and their respective factor loading values. Scores were then standardized around their means. The score consisted of three components: an ‘Enterocyte Integrity’ score that measured the physiological state of gut enterocytes and two inflammation scores that were classed as either, ‘Acute’, a measure of the innate neutrophil response or ‘Chronic’, a measure of the adaptive immune response.

Fisher’s Exact tests were used to test for significant differences in the proportion of positive samples (samples that tested positive for a pathogen) by age category and Wilcoxon Rank Sum tests were used to test for significant differences in pathogen gene counts between age categories. Correlation among biomarkers was assessed using Spearman correlation coefficients. Log-binomial models were used to determine the association between pathogen presence and 2-week diarrheal disease prevalence. The association between scores and stool pathogen gene counts was assessed using linear models. Models were adjusted for the infant’s sex, age, and the consistency of the stool sample. All analysis was done using R version 4.1.0. PCA’s were performed using the *FactoMineR* package.

## Results

### Descriptive Statistics

Our sample of 136 were from infants between 6 months to 2 years (mean = 14.35 months, SD = 4.80 months). Most were male (59%) and most were partially breastfed (77%). Only 7% of the infants were exclusively breastfed at the time of sampling. Most stool samples provided were formed (68%), with only 14% were classified as liquid.

The mean LAZ, WAZ, and WLZ of the infants in our sample were -0.89 (SD = 1.39), -0.23 (SD = 1.12), and 0.30 (SD = 1.20) respectively. Only 19, 6, and 2 infants had LAZ, WAZ, and WLZ scores ≤ -2 respectively (Table 1). A comparison of our biomarker values (Table 1) with previously reported studies is provided in S3–S10 Tables.

**Table 1:** Demographic characteristics and descriptive statistics of infants and biomarkers.

	N	Mean	SD	Minimum	Maximum	Median (25th, 75th percentiles)
LAZ	136	-0.89	1.39	-4.75	1.98	-0.89 (-1.90, 0.12)
WAZ	136	-0.23	1.12	-2.56	3.03	-0.38,(-1.03, 0.43)
WLZ	136	0.30	1.20	-2.44	3.95	0.14 (-0.62, 1.22)
Age, months	136	14.35	4.80	6.00	24.00	14.00 (11.00, 18.00)
MPO, ng/mL	134	7080.21	12130.41	137.40	102498.70	2970 (1382.9, 8563.4)
Neopterin, nmol/L	93	1509.34	1183.41	4.40	4635.60	1166.3 (576.1, 2176.4)
AAT, ng/mL	119	596.42	617.67	14.58	3915.67	411.64 (205.38, 753.33)
SI	136	2.62	24.94	0.00	288.18	0.027 (0.00, 0.087)
CDX1	136	0.10	0.12	0.00	0.69	0.070 (0.027, 0.13)
MUC12	136	4.71	6.29	0.00	41.15	2.34 (1.15, 5.52)
S100A8	136	10.68	20.55	0.04	183. 17	4.48 (2.23, 13.45)

### Stool pathogen frequency and gene counts in stool samples

**Fig 2:**
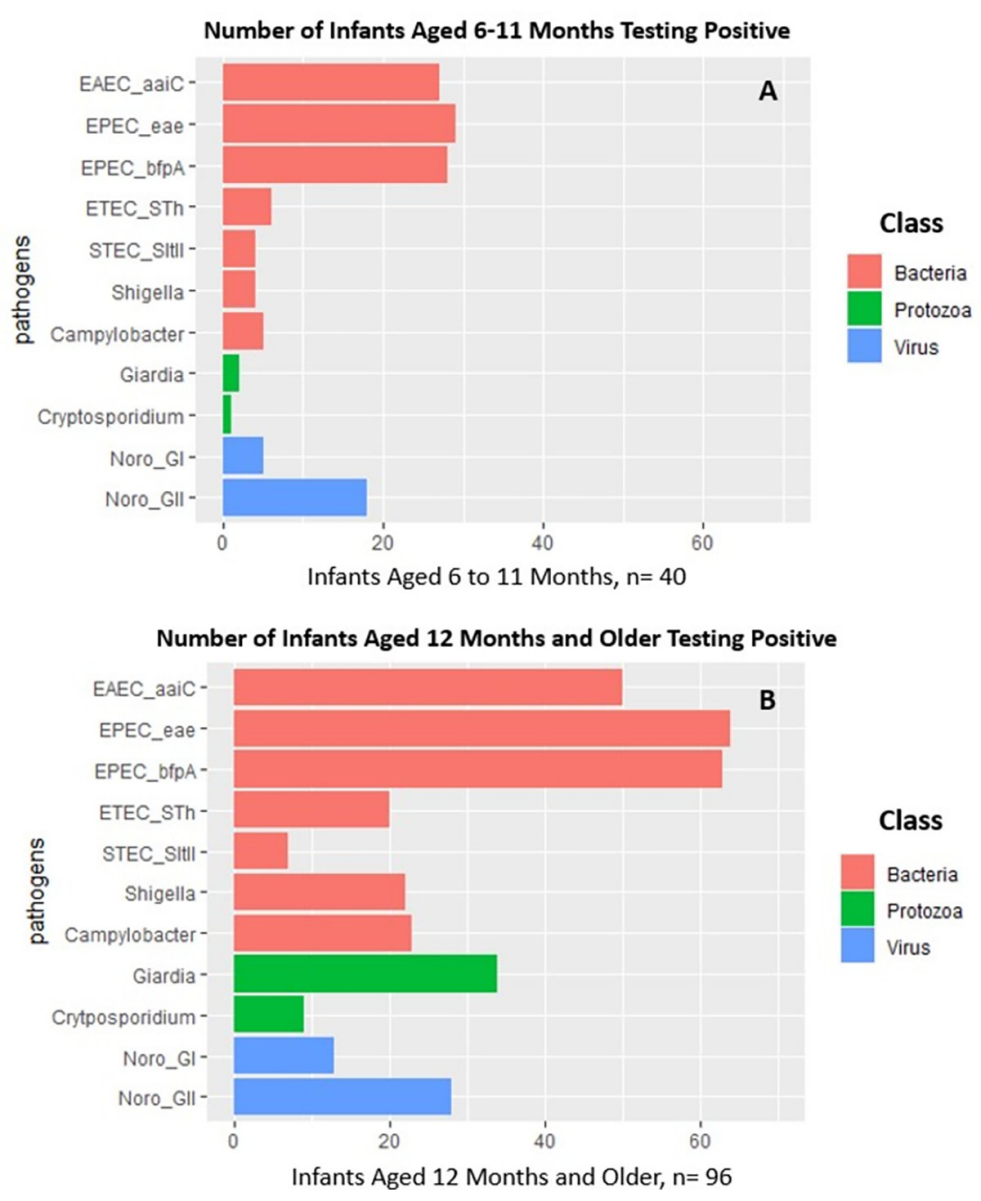
The number of infant stool samples testing positive for pathogenic gene markers in; (a) infants aged 6 to 11 months old (b), infants aged 12 months and older from informal settlements in Addis Ababa, Ethiopia.

Only the proportion of infants testing positive for *Giardia* was significantly different between the two age categories, with 5% of infants 6 to 11 months old testing positive compared to 35% of infants 12 months and older (p-value <0.001) (Fig 2 and S14 Table). Bacterial pathogens were the most frequently detected pathogen type in both age groups. For example, 73% of stool samples from infants 6 to 11 months old tested positive for the EPEC gene *eae*, as did 67% of stool samples from infants 12 months and older. However, there were no statistically significant differences in the proportion of infants testing positive for a bacterial pathogen between the two age categories. Norovirus GII was more commonly detected than norovirus GI, detected in 45% of infants aged 6 to 11 months old and in 29% of infants 12 months and older. As with bacterial pathogens, there were no statistically significant differences in the proportion of infants testing positive for either norovirus GI or GII between the two age categories.

Bacterial pathogens (specifically EAEC and EPEC) had the highest stool gene counts (Table 2). In general, EAEC and EPEC gene counts were highest in infants aged 6 to 11 months with marked decreases with age (p-value = 0.048 for EAEC). Norovirus GII stool gene counts were higher than those for Norovirus GI and were lower in infants aged 12 months and older. Among protozoa, *Giardia* stool gene counts were significantly higher in infants aged 12 months and older (p-value <0.001).

**Table 2:** Geometric means of pathogen stool gene counts by age category. Statistically significant differences in pathogen loads by age category are bolded.

	6 to 11 months (N = 40)	12 months and older (N = 96)	
**Pathogen**	**Mean (95% CI)**	**Mean (95% CI)**	**p-value**
**EAEC_aaiC (gene copies/g)**	**4.75E+03 (3.54E+02,6.36E+04)**	**2.55E+02 (4.99E+01, 1.30E+03)**	**<0.05**
EPEC_eae (gene copies/g)	1.15E+04(8.55E+02, 1.55E+05)	1.60E+03 (3.54+02, 7.28E+03)	0.12
EPEC_bfpA (gene copies/g)	7.20E+03 (5.09E+02, 1.02E+05)	1.37E+03 (2.97E+02, 6.31E+03)	0.17
ETEC_STh (gene copies/g)	6.03E+00 (1.39E+00, 2.62E+01)	9.57E+00 (3.64E+00, 2.52E+01)	0.46
STEC_SltII (gene copies/g)	3.67E+00 (1.48E+00, 9.53E+00)	2.77E+00 (1.75E+00, 4.40E+00)	0.59
*Shigella* (gene copies/g)	5.27E+00 (1.52E+00, 1.83E+01)	2.21E+01(7.48E+00, 6.53E+01)	0.10
*Campylobacter* (gene copies/g)	5.66E+00 (1.30E+00, 2.46E+01)	1.61E+01 (5.76E+00, 4.48E+01)	0.17
***Giardia* (gene copies/g)**	**1.89E+00 (6.50E-01, 5.48E+00)**	**7.26E+01 (2.05E+01, 2.57E+02)**	**<0.05**
*Cryptosporidium* (gene copies/g)	1.70E+00 (9.90E-01, 2.92E+00)	3.44E+00 (1.80E+00, 6.55E+00)	0.16
Noro_GI	5.50E-01 (1.40E-01, 2.16E+00)	7.50 E-01 (2.80E-01, 1.98E+00)	0.84
Noro_GII	2.99E+01 (3.78E+00, 2.37E+02)	4.37E+00 (1.42E+00, 1.35E+01)	0.09

### Spearman correlation coefficients between biomarkers

The strongest significant correlation between biomarkers was between MPO and AAT (Spearman Coefficient = 0.45) (Table 3). MPO also had weak significant correlations with S100A8 (Spearman Coefficient = 0.23) and mucin 12 (Spearman Coefficient = -0.20). The transcription factor, Cdx1, was also weakly correlated with SI (Spearman Coefficient = 0.30) and S100A8 (Spearman Coefficient = -0.17).

**Table 3:** Spearman correlation coefficients between biomarkers. Coefficients significant at the 0.05 level are bolded.

	SI	S100A8	MUC12	CDX1	AAT	MPO	Neopterin
SI	1.00						
S100A8	-0.02	1.00					
MUC12	0.07	0.17	1.00				
CDX1	**0.30**	**-0.17**	0.08	1.00			
AAT	-0.12	0.02	0.04	-0.06	1.00		
MPO	-0.15	**0.23**	**-0.20**	-0.07	**0.45**	1.00	
Neopterin	-0.01	-0.19	-0.20	0.04	0.07	0.02	1.00

### Principal Component Analysis (PCA)

The PCA analysis indicated that 5 components were required to explain the variability in the data (Table 4). Based on the biomarkers selected, each component represented a different physiological score. Two components represented chronic inflammation: **Chronic Inflammation A** consisting of SI (0.44), AAT (0.49), and neopterin (0.50); and **Chronic Inflammation B** consisting of Cdx1(-0.41), mucin 12 (-0.63), and neopterin (0.62). Two components represented acute inflammation: **Acute Inflammation A** consisting of Cdx1 (-0.40), S100A8 (0.46), and MPO (0.59); and **Acute Inflammation B** consisting of Cdx1 (0.68) and S100A8 (0.49). The fifth component represented **Enterocyte Integrity** consisting of SI (-0.59), mucin 12 (-0.47), and S100A8 (-0.49).

**Table 4:** Factor loading values and the derivation of PCA score models. Factor loading values of > 0.4 or < -0.4 are bolded.

	Full Panel PCA				
Biomarker	PC1: Chronic Inflammation A	PC2: Acute Inflammation A	PC3: Chronic Inflammation B	PC4: Enterocyte Integrity	PC5: Acute Inflammation B
MPO	0.32	**0.59**	-0.09	0.14	0.36
AAT	**0.49**	0.33	-0.09	0.28	-0.27
Neopterin	**0.50**	0.00	**0.62**	0.01	0.07
SI	**0.44**	-0.39	0.15	**-0.59**	0.12
CDX1	0.22	**-0.40**	**-0.41**	0.31	**0.68**
MUC12	0.29	0.11	**-0.63**	**-0.47**	-0.28
S100A8	-0.28	**0.46**	0.10	**-0.49**	**0.49**
Standard deviation	1.22	1.16	1.08	0.99	0.92
Proportion of Variance	0.21	0.19	0.17	0.14	0.12
Cumulative Proportion	0.21	0.40	0.57	0.71	0.83

### Associations between stool pathogen gene counts and the theory driven score

**Fig 3:**
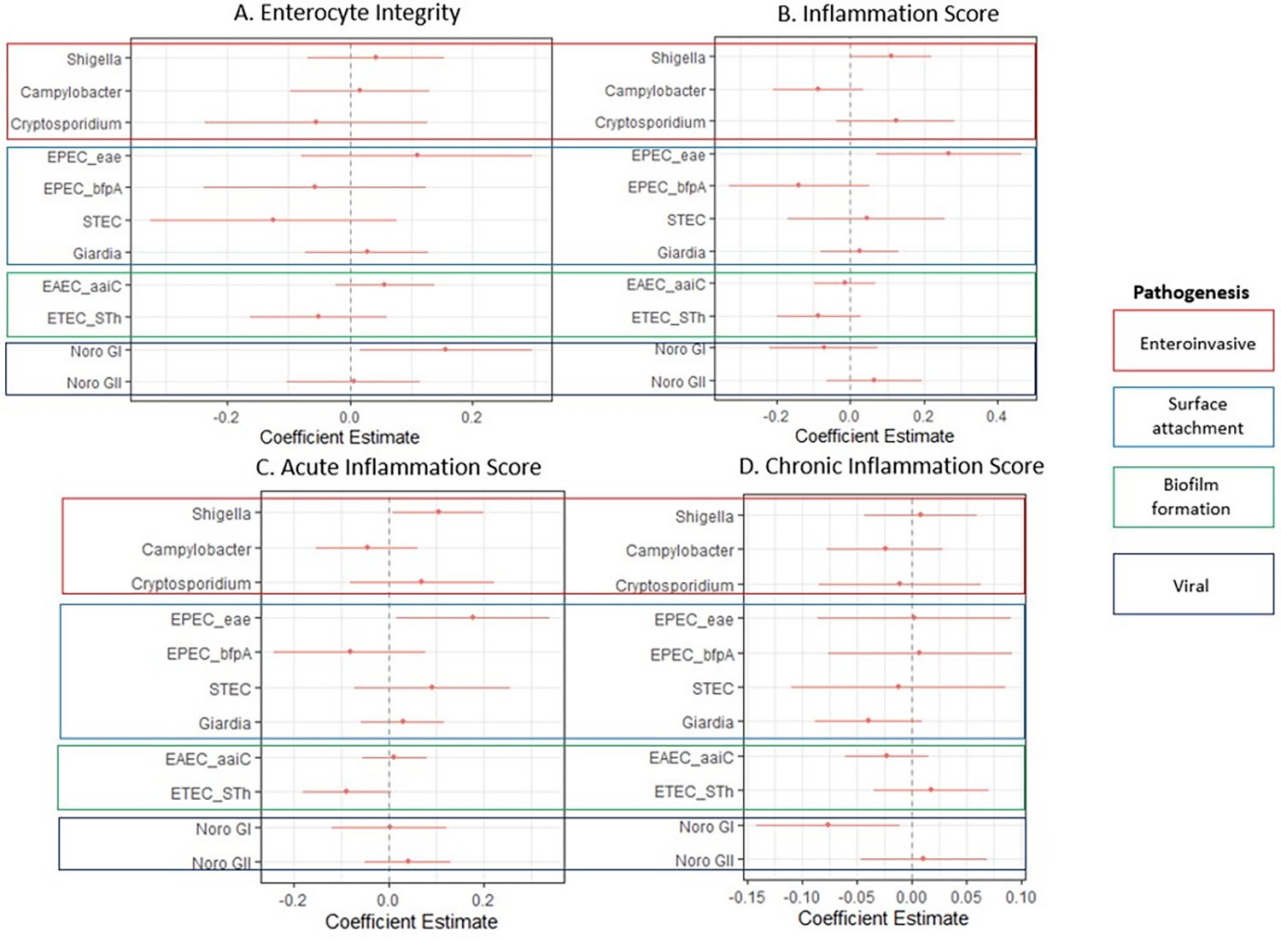
Associations between the theory driven score and stool pathogen gene counts; (a) associations between stool pathogen gene counts and the Enterocyte Integrity Score, (b) associations between the overall Inflammation Score and stool pathogen gene counts, (c) the associations between stool pathogen gene counts and Acute Inflammation, and (d) associations between the Chronic Inflammation Score and stool pathogen counts.

Multivariable linear models were used to examine association between stool pathogen gene counts and the three ‘theory driven’ scores (Fig 3). Noro GI was significantly associated with both the Enterocyte Integrity Score and the Chronic Inflammation Score, after adjusting for all stool pathogens, infant age, breast feeding status and stool consistency. *Shigella* was significantly associated with Acute Inflammation Score. Score summary statistics and age group stratified analysis are provided in S11 Table and S2 Fig respectively.

### Associations between stool pathogen gene counts and the data derived PCA score

*Shigella* stool gene counts were significantly associated with the Enterocyte Integrity Score as well as the Acute Inflammation Score B. ETEC_STh was significantly associated with the Acute Inflammation A score. Chronic Inflammation A and B scores were not significantly associated with any pathogen stool gene counts. Score summary statistics and age stratified analysis are provided in S12 Table and S3 Fig respectively.

## Discussion

Our findings add to the growing body of evidence that enteric pathogen infections and their hypothesized sequalae such as EED cannot be captured by a single catch-all definition but rather result in multiple overlapping responses to pathogen exposure, differing in time and, potentially, in physiological space [36]. Our results also confirm the findings of Manary and colleagues in demonstrating that fecal mRNA transcripts hold promise for the measurement of EED [20–23]. The utility of mRNA transcripts lies in their ability to measure specific cellular processes, providing a more complete picture of the overall gut state. Given the time scales that mRNA transcripts measure, they provide a more immediate snapshot of physiological and immune responses. This may be especially valuable in understanding dynamic processes such as pathogen infection and colonization. Given recent efforts to develop ‘panels’ of EED biomarkers [37,38], coupling panels with metabolic and transcriptomic approaches will be especially valuable.

**Fig 4:**
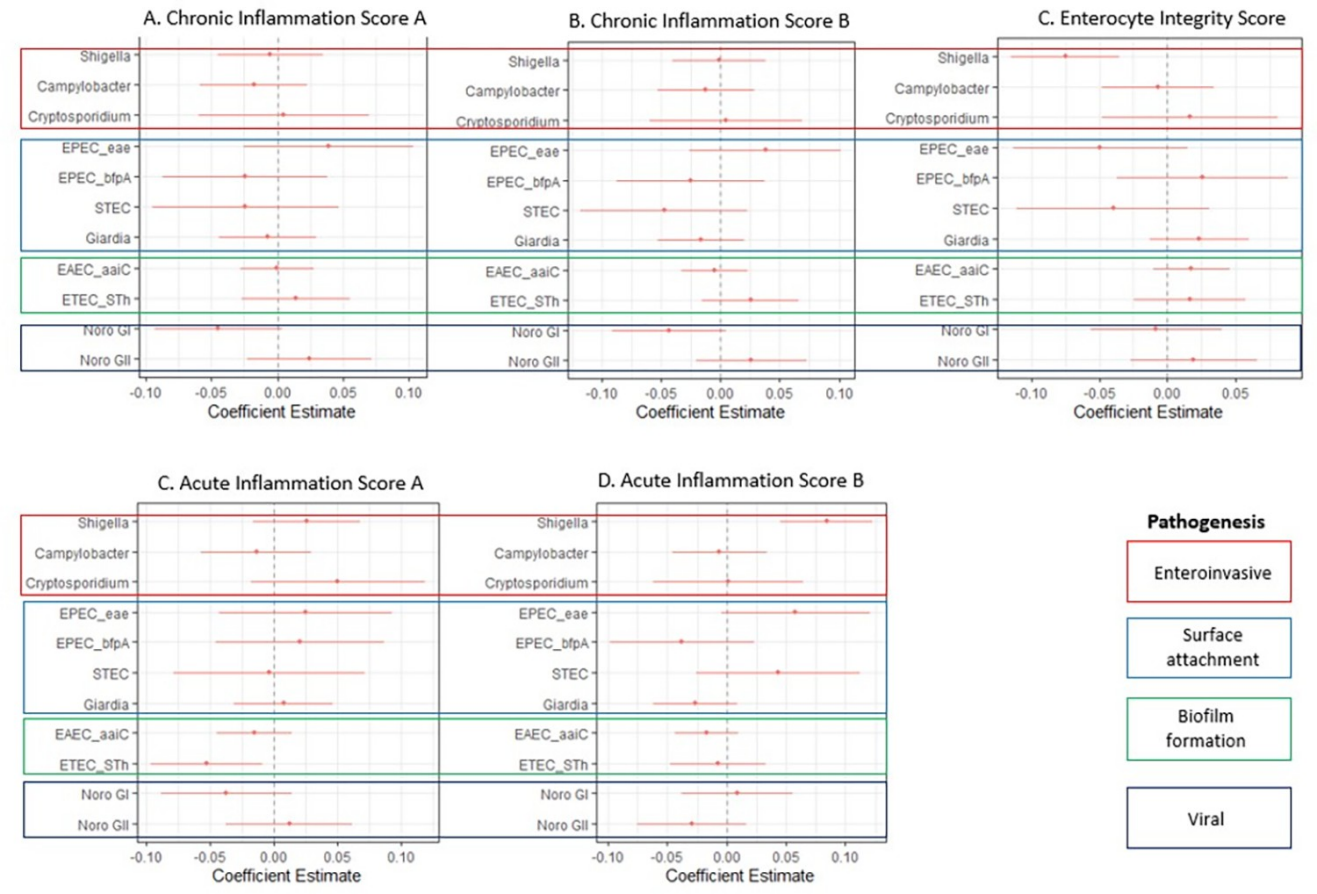
Associations between the data derived score and stool pathogen gene counts; (a) associations between Chronic Inflammation Score A (SI, AAT, and neopterin) and stool pathogen gene counts, (b) associations between Chronic Inflammation Score B (Cdx1, mucin12, and neopterin) and stool pathogen gene counts (c) associations between Enterocyte Integrity (SI, mucin12, and S100A8) and stool pathogen gene counts, (d) associations between stool pathogen gene counts and the Acute Inflammation Score A (Cdx1, S100A8, and MPO), and (e) associations between stool pathogen gene counts and Acute Inflammation Score B (Cdx1 and S100A8).

The use of two different scoring systems demonstrates that there are multiple ways to capture underlying pathogenic processes and scoring systems can be adapted to best suit the goals of a study. Our ‘theory driven’ and ‘data derived’ (including a data derived score using only MPO, AAT and, neopterin (See S13 Table and S4 Fig)) scores largely agree especially regarding how they capture the associations between stool pathogen gene counts and acute and chronic inflammation. Notably, both approaches demonstrate how *Shigella* infection not only elicits a strong neutrophil response (Figs 3, 4, and S4), but also profoundly affects enterocyte integrity as shown by the negative association between *Shigella* gene stool gene counts and the Enterocyte Integrity Score in the “data derived” score (Fig 4). We believe that the biomarkers detect the massive inflammatory response associated with apoptotic macrophages, infiltration of polymorphonuclear leukocytes and epithelial destruction that are characteristic of shigellosis[13]. Our ability to capture the impact of *Shigella* infection on enterocyte integrity is due to the inclusion of the mRNA transcript biomarkers which provide cell specific readouts, which in the case of shigellosis is the destruction of gut enterocytes. Strikingly, however, the enterocyte integrity scores did not agree between the two scoring systems. Given the complexity of EED as well as the poorly understood and complex relationships between the different biomarkers, the “theory driven” score may have subsumed multiple etiologic processes into a single biomarker, especially when using a small biomarker panel and sample.

Our work also shows that pathogenic *E*. *coli* carriage may be the norm in highly contaminated settings. This is similar to other studies that have reported that a significant proportion of children continue to have sub-clinical pathogen carriage [39–42]. EPEC, the most detected pathogen, was associated with both acute and chronic inflammation processes. EPEC infection results in localized destruction of the intestinal brush border and distortion of the apical enterocyte membrane [43]. EPEC infection has been proposed to occur in four stages: 1) EPEC cells express 3 proteins (bundle-forming pili (Bfp), intimate adhesin intimin, and short surface-associated filaments (EspA filaments) [43]; 2) EPEC cells adhere to epithelial cells via Bfp and EspA filaments and cause depolymerization of actin and the loss of microvilli [43]; 3) EspA filaments are lost from the bacterial cell surface and adhesin binding results in the accumulation of actin and other cytoskeletal elements beneath the site of bacterial adherence [43]; 4) bacterial attachment results in massive accumulation of cytoskeletal elements resulting in the loss of tight-junction integrity and mitochondrial function that leads to both electrolyte loss and eventual cell death [43]. Future research could focus on whether biomarker levels are associated with different stages of EPEC infection.

The prolonged and asymptomatic carriage of pathogens such as EPEC, necessitates the need for diagnostic techniques that can distinguish between symptomatic and sub-clinical infections. Though we did not see any significant differences in scores between infants who had diarrhea in the past 2-weeks and those who had not (S15 Table), our work shows that there are measurable physiological processes associated with infection, regardless of symptom presentation. For example, though the detection of *Shigella* was not associated with 2-week diarrheal disease prevalence, we were able to measure physiological impacts of *Shigella* infection, including enterocyte damage and acute inflammation. We hope that future work can further explore how curated biomarker panels can be used to distinguish between symptomatic and sub-clinical infections, which the small sample size and cross-sectional nature of our study precluded us from doing.

To our knowledge, no other studies have characterized mRNA transcript-based biomarkers in an urban population of infants. Compared to previously reported scores from rural populations of infants in Malawi and Sierra Leone, our mRNA transcript expression levels would suggest better gut health in our infants (S6–S10 Tables). Fecal protein biomarker measurements on the hand were in broad agreement with what has previously been reported (S3–S5 Tables). Mean biomarker levels for MPO and neopterin, are marginally lower compared to other studies, while AAT levels were similar (7080.21ng/mL, 1509.34nmol/L, and 596.42 ng/mL respectively). AAT is a modulator of the neutrophil response and inhibits MPO, and the higher ratio of AAT to MPO in our sample compared to that reported in other studies, may be indicative of a more controlled immune response in our samples [44]. The differences in agreement between the mRNA transcript and stool protein markers suggests that mRNA transcripts and protein biomarkers may measure responses that differ physiologically and temporally. Though at the protein level infants appeared broadly similar to populations reported elsewhere [9,15,45–47], the differences between our study and previously reported studies at the transcript level may indicate that responses to stressors vary by geography and population even though the end result is broad systemic inflammation. Specific biomarker panels to measure both specific cellular and physiologic processes and relevant EED endpoints will be especially valuable in the establishing causality of the inflammatory end-states. Recent findings, such the establishment of a causal relationship between the duodenal microbiota and growth faltering when combined with metabolomics should aid biomarker selection, both at the mRNA transcript and protein levels [6].

Overall, our findings add to a growing body of literature showing that the evaluation of physiological processes resulting in systemic inflammatory endpoints will require more nuanced approaches and that a standard set of diagnostic criteria may be hard to define. Though the selected biomarkers could broadly measure physiological and immunological processes, a more detailed understanding of the continuum of physiological responses ranging from transient immune responses to chronic systemic dysregulation is necessary. In addition, given that a goal of our study was to evaluate whether biomarker panels can measure gut specific processes, we did not specifically evaluate inter-biomarker associations beyond correlations. Future work that addresses some of our limitations such as small sample size and lack of temporal follow up, will be needed to better evaluate any relationships between biomarkers. The development of biomarker panels can provide valid measures of overall gut health, but also to provide cell-specific measures that inform our understanding of the physiological responses to transient insults, long-term asymptomatic pathogen carriage, and chronic end states such as EED. Such panels are valuable tools that complement existing direct measures of EED such as the L:M test in understanding the impact of pathogenic stressors on long-term outcomes in infants in low resource settings.

## Supporting information

S1 Text Droplet Digital PCR Detection of mRNA Transcripts. Protocol for the detection of mRNA transcripts using ddPCR.
(DOCX)

S2 Text Droplet Digital PCR Quantification of Stool Pathogen Loads. Protocol for the quantification of stool pathogen gene loads using ddPCR.
(DOCX)

S1 Table Pathogen specific gene targets used for the quantification of stool pathogen loads. Gene targets and ddPCR Supermix used for the quantification of stool pathogen gene loads.
(DOCX)

S2 Table Derivation of the theory driven histological score based on indicators laid out in Liu et.al (2020). Explanation of the derivation of the theory driven score.
(DOCX)

S3 Table Comparison of study myeloperoxidase levels with previously reported levels. Table comparing study measured myeloperoxidase levels with levels reported in a subset of previous studies.
(DOCX)

S4 Table Comparison of study AAT levels with previously reported levels. Table comparing study measured AAT levels with levels reported in a subset of previous studies.
(DOCX)

S5 Table Comparison of study neopterin levels with previously reported levels. Table comparing study measured neopterin levels with levels reported in a subset of previous studies.
(DOCX)

S6 Table Comparison of study transcript expression levels and expression levels previously reported in Malawian infants with varying L:M ratios. Table comparing study transcript levels with transcript levels from Malawian infants with different L:M ratios.
(DOCX)

S7 Table Comparison of study transcript expression levels previously reported expression levels in Malawian infants. Table comparing study transcript levels with transcript levels in two previous studies in Malawian infants.
(DOCX)

S8 Table Comparison of study transcript expression levels with expression levels in Malawian infants aged less than 12 months and 12–61 months. Comparison of study transcript levels with age stratified transcript levels from Malawian infants and young children.
(DOCX)

S9 Table Comparison of study transcript expression levels with those of Malawian infants with severe EED and no to moderate EED. Comparison of study transcript levels with levels in Malawian infants with and without EED.
(DOCX)

S10 Table Comparison of study transcript expression levels with those of Sierre Leonean Infants. Comparison of study transcript levels with transcript levels in Sierra Leonean infants.
(DOCX)

S11 Table Summary statistics of the theory driven scores. (DOCX)

S12 Table Summary statistics of the data derived scores. (DOCX)

S13 Table Factor loading values and score types for PCA derived using only myeloperoxidase, AAT and neopterin. (DOCX)

S14 Table Number of samples testing positive for each pathogen. (DOCX)

S15 Table Comparison of Scores by 2-week diarrheal disease prevalence. (DOCX)

S16 Table Unadjusted associations between pathogen prevalence and 2-week diarrheal disease prevalence. (DOCX)

S1 Fig Correlation between age and biomarker levels in the full sample. (PDF)

S2 Fig Associations between the theory driven score and age stratified stool pathogen gene counts. (PDF)

S3 Fig Associations between the data derived score and age stratified stool pathogen gene counts. (PDF)

S4 Fig Associations between the protein biomarker-based data derived score and stool pathogen gene counts. (PDF)

S5 Fig Associations between the protein biomarker-based data derived score and stool pathogen gene counts in a) infants aged 6–11 months and b) in infants aged 12 months and older. (PDF)
