# Supplementary material for: Stool biomarkers as measures of enteric pathogen infection in infants from Addis Ababa informal settlements
Source: PLoS Negl Trop Dis. 2023 Feb 21;17(2):e0011112. doi: 10.1371/journal.pntd.0011112 (PMC9983878; doi:10.1371/journal.pntd.0011112)
Supplement: S1 Text — Protocol for the detection of mRNA transcripts using ddPCR. (DOCX) [file pntd.0011112.s001.docx]

**S1 Text: Droplet Digital PCR Detection of mRNA Transcripts**

Assays were setup using the One-Step RT-ddPCR Supermix (Bio-Rad, Hercules, CA) containing 5ul Supermix, 2ul Reverse Transcriptase, DTT at 15mM, 1ul each of each 20 x TaqMan Gene Expression Assay (Applied Biosystems, Carlsbad, CA) and 60ng of template or sterilized water to bring the reaction volume to 20ul. Droplets were generated using the QX200 AutoDG Droplet Digital PCR system (Bio-Rad, Hercules, CA). The generated droplets (40 ul) were loaded into a 96-well plate and sealed using a PX1 plate sealer (Bio-Rad, Hercules, CA). Thermocycling conditions consisted of 60 min reverse transcription at 50 °C, enzyme activation for10 min at 95 °C, followed by 40 cycles of denaturation at 94 °C for 30 s, annealing and extension at 60°C for 1 min; followed by enzyme deactivation at 98°C for 10 min, and a continuous hold at 4°C. All samples were run on the C1000 Touch thermocycler (Bio-Rad, Hercules, CA). Glyceraldehyde-3-phosphate dehydrogenase (GAPDH) reactions were run separately for each sample for normalization of targets.
