# Supplementary material for: Stool biomarkers as measures of enteric pathogen infection in infants from Addis Ababa informal settlements
Source: PLoS Negl Trop Dis. 2023 Feb 21;17(2):e0011112. doi: 10.1371/journal.pntd.0011112 (PMC9983878; doi:10.1371/journal.pntd.0011112)
Supplement: S2 Text — Protocol for the quantification of stool pathogen gene loads using ddPCR. (DOCX) [file pntd.0011112.s002.docx]

**S2 Text: Droplet Digital PCR Quantification of Stool Pathogen Loads**

DNA assays were setup by combining 10ul of ddPCR Supermix for Probes (no dUTP), primers at 900nM, probes at 250 nM, and 60ng of template DNA or sterilized water for no template controls. RNA assays were setup using the One-Step RT-ddPCR Supermix (Bio-Rad, Hercules, CA) containing 5ul Supermix, 2ul Reverse Transcriptase, DTT at 15mM, primers at 900nM, probes at 250nM and 60ng of template RNA or sterilized water for no template controls. Droplets were generated using the QX200 AutoDG Droplet Digital PCR system (Bio-Rad, Hercules, CA). The generated droplets (40ul) were loaded into a 96-well plate and sealed using a PX1 plate sealer (Bio-Rad, Hercules, CA). Next the following 3 steps were taken: 1) For 40 cycles, DNA thermocycling conditions consisted of enzyme activation at 95°C for 10 min; denaturation at 94C for 30 s; annealing and extension at 58°C for 1 min. 2) Enzyme deactivation occurred at 98°C for 10 min; and 3) and a continuous hold at 4°C. RNA thermocycling conditions consisted of 1) 60 min reverse transcription at 50 °C, 2) enzyme activation for 10 min at 95 °C, 3) 40 cycles of denaturation at 94 °C for 30 s, 4) annealing and extension at 58°C for 1 min; 5) enzyme deactivation at 98°C for 10 min, and 6) a continuous hold at 4°C. All samples were run on the C1000 Touch thermocycler (Bio-Rad, Hercules, CA), with a lid temperature of 105°C, a sample volume of 40ul and a ramp rate of 2°C. On completion of the thermocycling, plates were read using the QX200 Droplet Reader (Bio-Rad, Hercules, CA, USA) and QuantaSoft software (Bio-Rad, Hercules, CA).
