# Supplementary material for: Stool biomarkers as measures of enteric pathogen infection in infants from Addis Ababa informal settlements
Source: PLoS Negl Trop Dis. 2023 Feb 21;17(2):e0011112. doi: 10.1371/journal.pntd.0011112 (PMC9983878; doi:10.1371/journal.pntd.0011112)
Supplement: S1 Table — Gene targets and ddPCR Supermix used for the quantification of stool pathogen gene loads. (DOCX) [file pntd.0011112.s003.docx]

**S1 Table: Pathogen specific gene targets used for the quantification of stool pathogen loads.**

| **Pathogen** | **Gene Target** | **Supermix** |
| --- | --- | --- |
| EAEC | *aaiC* | ddPCR Supermix for Probes (No dUTP) |
| EPEC | *Eae, bfpA* | ddPCR Supermix for Probes (No dUTP) |
| ETEC | *STh* | ddPCR Supermix for Probes (No dUTP) |
| STEC | *SltII* | ddPCR Supermix for Probes (No dUTP) |
| *Shigella* | *ipaH* | ddPCR Supermix for Probes (No dUTP) |
| *Campylobacter* | *cadF* | ddPCR Supermix for Probes (No dUTP) |
| *Giardia* | *18S rRNA* | ddPCR Supermix for Probes (No dUTP) |
| *Cryptosporidium* | *18S rRNA* | ddPCR Supermix for Probes (No dUTP) |
| Norovirus GI | *ORF 1-2* | 1-Step RT-ddPCR Advanced Kit for Probes |
| Norovirus GII | *ORF 1-2* | 1-Step RT-ddPCR Advanced Kit for Probes |
