# Supplementary material for: Stool biomarkers as measures of enteric pathogen infection in infants from Addis Ababa informal settlements
Source: PLoS Negl Trop Dis. 2023 Feb 21;17(2):e0011112. doi: 10.1371/journal.pntd.0011112 (PMC9983878; doi:10.1371/journal.pntd.0011112)
Supplement: S2 Table — Explanation of the derivation of the theory driven score. (DOCX) [file pntd.0011112.s004.docx]

**S2 Table:** **Derivation of the theory driven histological score based on indicators laid out in Liu et.al (2020)**[1]**.**

| **Biomarker** | **Liu *et al.* (2020) Indicators** | **Grading Scheme** | **Justification** |
| --- | --- | --- | --- |
| Sucrase Isomaltase | Villus Architecture | Quintiles of SI levels with highest quintile corresponding to Grade 0 and lowest to Grade 4 | Sucrase Isomaltase (SI) is located on the brush border membrane (BBM) of the intestinal epithelium where it is involved in the final step of starch digestion. SI is produced in the cytoplasm of epithelial cells before being transported and localized to the BBM surface. SI levels should be indicators of overall enterocyte structure and function |
|  | Gestalt Architecture |  |  |
|  | Enterocyte Injury |  |  |
| AAT | Epithelial Detachment | Quintiles of AAT levels with the highest quintile corresponding to Grade 0 and the lowest to Grade 4 | AAT is a measure of disturbed barrier function related to intestinal inflammation. The transmigration of neutrophils through the epithelial barrier promotes the release of host defense proteins eventually resulting in mucosal damage from chronic inflammation. The breakdown of epithelial barrier function results in the presence of serum proteins such as AAT in stool. |
|  | Paneth Cell Density |  |  |
| MUC12 | Enterocyte Injury | Quintiles of Mucin 12 expression with the highest quintile corresponding to Grade 0 and the lowest to Grade 4 | MUC12 is part of the transmembrane mucins that forms the glycocalyx and is produced by enterocytes. MUC12 expression decreases with enterocyte injury and should track with SI and CDX1. |
| CDX1 | Enterocyte Injury | Quintiles of CDX1 expression with the highest quintile corresponding to Grade 0 and the lowest to Grade 4 | CDX1 is closely tied to enterocyte structure and may be involved in the transcriptional regulation of SI |
| Neopterin | Intraepithelial Lymphocytes | Quintiles of Neopterin expression with the highest quintile corresponding to Grade 0 and the lowest to Grade 4 | Neopterin is a good marker of general inflammation as well as the presence of activated immune cells |
|  | Chronic Inflammation of Lamina Propria |  |  |
| MPO | Acute Inflammation | Quintiles of MPO expression with the highest quintile corresponding to Grade 0 and the lowest to Grade 4 | MPO is marker of the short term rapid innate immune response which typically represents the first of line of defense against pathogens and injury. MPO is secreted by neutrophils |
| S100A8 | Eosinophil Infiltration | Quintiles of S100A8 expression with the highest quintile corresponding to Grade 0 and the lowest to Grade 4 | S100A8 is also a marker of acute inflammation and is secreted by both neutrophils and eosinophils depending on the healing/injury state. |

**References**

1. Liu TC, Vanbuskirk K, Ali SA, Kelly MP, Holtz LR, Yilmaz OH, et al. A novel histological index for evaluation of environmental enteric dysfunction identifies geographic-specific features of enteropathy among children with suboptimal growth. PLoS Negl Trop Dis. 2020;14: 1–21. doi:10.1371/journal.pntd.0007975
