# Supplementary material for: Stool biomarkers as measures of enteric pathogen infection in infants from Addis Ababa informal settlements
Source: PLoS Negl Trop Dis. 2023 Feb 21;17(2):e0011112. doi: 10.1371/journal.pntd.0011112 (PMC9983878; doi:10.1371/journal.pntd.0011112)
Supplement: S3 Table — Table comparing study measured myeloperoxidase levels with levels reported in a subset of previous studies. (DOCX) [file pntd.0011112.s005.docx]

**S3 Table:** **Comparison of study myeloperoxidase levels with previously reported levels.**

| **Biomarker** | **Data Set** | **N** | **Mean +/- SD** | **Min** | **Max** | **Median (25th, 75th percentiles)** |
| --- | --- | --- | --- | --- | --- | --- |
| MPO(ng/mL) | **Ethiopian Infants** | **134** | **7080.21 +/- 12130.41** | **137.4** | **102498.7** | **2970 (1382.9, 8563.4)** |
|  | Singh *et al.* (2021)[1] | 200 | - | - | - | 42172.51(19253.97,87815.40) |
|  | Colston *et al.* (2017)[2] | 4064 | 12482.8 | 0 | 111145.7 | - |
|  | Campbell *et al.* (2017)[3] | 498 | 4460.3 (4145.0, 4799.5)* |  |  |  |
|  | Arndt *et al.* (2016)[4] | 1185 |  |  |  | 3354.9 (1594.9, 7430.1) |
|  | Kosek *et al.* (2013)-Bangladesh[5] | - | - | - | - | 8838.23 (4552.95, 15020.98) |
|  | Kosek *et al.* (2013)-Brazil[5] | - | - | - | - | 6847.91 (3884.41, 12452.19) |
|  | Kosek *et al.* (2013)-India[5] | - | - | - | - | 14574.97 (6093.03, 27507.40) |
|  | Kosek *et al.* (2013)-Nepal[5] | - | - | - | - | 14484.40 (7499.47, 25317.29) |
|  | Kosek *et al.* (2013)-Peru[5] | - | - | - | - | 11623.52 (5765.75, 21883.94) |
|  | Kosek *et al.* (2013)-Pakistan[5] | - | - | - | - | 8452.01 (3950.82, 12868.78) |
|  | Kosek *et al.* (2013)-South Africa[5] | - | - | - | - | 16284.92 (6530.56, 25171.11) |
|  | Kosek *et al.* (2013)-Tanzania[5] | - | - | - | - | 17949.77 (9612.64, 2633.81) |
|  | Kosek *et al.* (2013)-Overall[5] | - | - | - | - | 11118.88 (5650.46, 20526.33) |
|  |  |  | * GM (95% CI) |  |  |  |

**References**

1. Singh A, Ghosh S, Ward H, Manary MJ, Rogers BL, Rosenberg IH. Biomarkers of environmental enteric dysfunction are differently associated with recovery and growth among children with moderate acute malnutrition in Sierra Leone. Am J Clin Nutr. 2021;113: 1556–1564. doi:10.1093/ajcn/nqaa434

2. Colston JM, Yori PP, Colantuoni E, Moulton LH, Ambikapathi R, Lee G, et al. A methodologic framework for modeling and assessing biomarkers of environmental enteropathy as predictors of growth in infants: An example from a Peruvian birth cohort. Am J Clin Nutr. 2017;106: 245–255. doi:10.3945/ajcn.116.151886

3. Campbell RK, Schulze KJ, Shaikh S, Mehra S, Ali H, Wu L, et al. Biomarkers of Environmental Enteric Dysfunction among Children in Rural Bangladesh. J Pediatr Gastroenterol Nutr. 2017;65: 40–46. doi:10.1097/MPG.0000000000001557

4. Arndt MB, Richardson BA, Ahmed T, Mahfuz M, Haque R, John-Stewart GC, et al. Fecal markers of environmental enteropathy and subsequent growth in Bangladeshi children. Am J Trop Med Hyg. 2016;95: 694–701. doi:10.4269/ajtmh.16-0098

5. Kosek M, Haque R, Lima A, Babji S, Shrestha S, Qureshi S, et al. Fecal Markers of Intestinal Inflammation and Permeability Associated with the Subsequent Acquisition of Linear Growth Deficits in Infants. Am J Trop Med Hyg. 2013;88: 390–396. doi:10.4269/ajtmh.2012.12-0549
