# Supplementary material for: Stool biomarkers as measures of enteric pathogen infection in infants from Addis Ababa informal settlements
Source: PLoS Negl Trop Dis. 2023 Feb 21;17(2):e0011112. doi: 10.1371/journal.pntd.0011112 (PMC9983878; doi:10.1371/journal.pntd.0011112)
Supplement: S6 Table — Table comparing study transcript levels with transcript levels from Malawian infants with different L:M ratios. (DOCX) [file pntd.0011112.s008.docx]

**S6 Table: Comparison of study transcript expression levels and expression levels previously reported in Malawian infants with varying L:M ratios.**

| **Transcript** | **Ethiopian Infants** | | **Agapova *et al.* (2013)**[1] | | | |
| --- | --- | --- | --- | --- | --- | --- |
|  |  |  | **Normal L:M** | | **Increased L:M** | |
|  | **Mean** | **Median (25th, 75th percentiles)** | **Mean** | **Median** | **Mean** | **Median** |
| SI | 2.62 | 0.027 (0.00, 0.087) | 0.0086 | 0.0103 | 0.008 | 0.5598 |
| Cdx1 | 0.10 | 0.070( 0.027, 0.13) |  |  |  |  |
| S100A8 | 4.71 | 2.34 (1.15, 5.52) | 0.7252 | 1.5854 | 0.2747 | 0.4888 |
| Mucin 12 | 10.68 | 4.48 (2.23, 13.45) |  |  |  |  |

**References**

1. Agapova S, Stephenson K, Manary M, Weisz A, Tarr PI, Mkakosya R, et al. Detection of low-concentration host mRNA transcripts in Malawian children at risk for environmental enteropathy. J Pediatr Gastroenterol Nutr. 2013;56: 66–71. doi:10.1097/MPG.0b013e31826a107a
