# Supplementary material for: Stool biomarkers as measures of enteric pathogen infection in infants from Addis Ababa informal settlements
Source: PLoS Negl Trop Dis. 2023 Feb 21;17(2):e0011112. doi: 10.1371/journal.pntd.0011112 (PMC9983878; doi:10.1371/journal.pntd.0011112)
Supplement: S7 Table — Table comparing study transcript levels with transcript levels in two previous studies in Malawian infants. (DOCX) [file pntd.0011112.s009.docx]

**S7 Table: Comparison of study transcript expression levels previously reported expression levels in Malawian infants.**

| **Transcript** | **Ethiopian Infants** | | **Stauber *et al.* (2016)**[1] | **Ordiz *et al.* (2016)**[2] | |  |
| --- | --- | --- | --- | --- | --- | --- |
|  |  |  |  |  |  |  |
|  | **Mean** | **Median (25th, 75th percentiles)** | **Median (25th, 75th percentiles)** | **Mean** | **Median (25th, 75th percentiles)** |  |
| SI | 2.62 | 0.027 (0.00, 0.087) | 0.017 (0.008, 0.036) | 0.114 | 0.017 (0.008, 0.036) |  |
| Cdx1 | 0.10 | 0.070( 0.027, 0.13) | 0.026 (0.016, 0.042) | 0.047 | 0.027 (0.016, 0.042) |  |
| S100A8 | 4.71 | 2.34 (1.15, 5.52) | 0.386 (0.154, 1.169) | 0.979 | 0.386 (0.154, 1.169) |  |
| Mucin 12 | 10.68 | 4.48 (2.23, 13.45) | 0.321 (0.162, 0.541) | 0.447 | 0.294 (0.163, 0.539) |  |

**References**

1. Stauber J, Shaikh N, Ordiz MI, Tarr PI, Manary MJ. Droplet digital PCR quantifies host inflammatory transcripts in feces reliably and reproducibly. Cell Immunol. 2016;303: 43–49. doi:10.1016/j.cellimm.2016.03.007

2. Ordiz MI, Shaikh N, Trehan I, Maleta KM, Stauber J, Shulman R, et al. Environmental Enteric Dysfunction Is Associated With Poor Linear Growth and Can Be Identified by Host Fecal mRNAs. J Pediatr Gastroenterol Nutr. 2016;63: 453–459. doi:10.1097/MPG.0000000000001315
