# Supplementary material for: Stool biomarkers as measures of enteric pathogen infection in infants from Addis Ababa informal settlements
Source: PLoS Negl Trop Dis. 2023 Feb 21;17(2):e0011112. doi: 10.1371/journal.pntd.0011112 (PMC9983878; doi:10.1371/journal.pntd.0011112)
Supplement: S8 Table — Comparison of study transcript levels with age stratified transcript levels from Malawian infants and young children. (DOCX) [file pntd.0011112.s010.docx]

**S8 Table:** **Comparison of study transcript expression levels with expression levels in Malawian infants aged less than 12 months and 12-61 months.**

| **Transcript** | **Ethiopian Infants** | | **Ordiz *et al.* (2018)**[1] | | | |
| --- | --- | --- | --- | --- | --- | --- |
|  |  |  | **Children <12 months** | | **Children 12–61 months** | |
|  | **Mean** | **Median (25^th^, 75^th^ percentiles)** | **Mean** | **Median (25^th^, 75^th^ percentiles)** | **Mean** | **Median (25^th^, 75^th^ percentiles)** |
| SI | 2.62 | 0.027 (0.00, 0.087) |  |  |  |  |
| Cdx1 | 0.10 | 0.070( 0.027, 0.13) | 0.024 | 0.018 (0.012, 0.029) | 0.047 | 0.027 (0.016, 0.042) |
| S100A8 | 4.71 | 2.34 (1.15, 5.52) | 1.927 | 1.406 (0.472, 2.304) | 0.979 | 0.386 (0.154, 1.169) |
| Mucin 12 | 10.68 | 4.48 (2.23, 13.45) | 0.351 | 0.217 (0.121, 0.450) | 0.447 | 0.294 (0.163, 0.539) |

**References**

1. Ordiz MI, Wold K, Kaimila Y, Divala O, Gilstrap M, Lu HZ, et al. Detection and interpretation of fecal host mRNA in rural Malawian infants aged 6–12 months at risk for environmental enteric dysfunction. Exp Biol Med. 2018;243: 985–989. doi:10.1177/1535370218794418
