# Supplementary material for: Stool biomarkers as measures of enteric pathogen infection in infants from Addis Ababa informal settlements
Source: PLoS Negl Trop Dis. 2023 Feb 21;17(2):e0011112. doi: 10.1371/journal.pntd.0011112 (PMC9983878; doi:10.1371/journal.pntd.0011112)
Supplement: S9 Table — Comparison of study transcript levels with levels in Malawian infants with and without EED. (DOCX) [file pntd.0011112.s011.docx]

**S9 Table: Comparison of study transcript expression levels with those of Malawian infants with severe EED and no to moderate EED.**

| **Transcript** | **Ethiopian Infants** | | **Ordiz *et al.* (2018)**[1] | | | |
| --- | --- | --- | --- | --- | --- | --- |
|  |  |  | **Severe EED** | | **Children no or moderate EED** | |
|  | **Mean** | **Median (25^th^, 75^th^ percentiles)** | **Mean** | **Median (25^th^, 75^th^ percentiles)** | **Mean** | **Median (25^th^, 75^th^ percentiles)** |
| SI | 2.62 | 0.027 (0.00, 0.087) |  |  |  |  |
| Cdx1 | 0.10 | 0.070( 0.027, 0.13) | 0.02 | 0.018 (0.010, 0.025) | 0.027 | 0.019 (0.013, 0.031) |
| S100A8 | 4.71 | 2.34 (1.15, 5.52) | 2.09 | 1.33 (0.38, 2.36) | 2.68 | 1.10 (0.50, 2.55) |
| Mucin 12 | 10.68 | 4.48 (2.23, 13.45) | 0.306 | 0.192 (0.121, 0.393) | 0.395 | 0.217 (0.123, 0.473) |

**References**

1. Ordiz MI, Wold K, Kaimila Y, Divala O, Gilstrap M, Lu HZ, et al. Detection and interpretation of fecal host mRNA in rural Malawian infants aged 6–12 months at risk for environmental enteric dysfunction. Exp Biol Med. 2018;243: 985–989. doi:10.1177/1535370218794418
