# Supplementary material for: Stool biomarkers as measures of enteric pathogen infection in infants from Addis Ababa informal settlements
Source: PLoS Negl Trop Dis. 2023 Feb 21;17(2):e0011112. doi: 10.1371/journal.pntd.0011112 (PMC9983878; doi:10.1371/journal.pntd.0011112)
Supplement: S10 Table — Comparison of study transcript levels with transcript levels in Sierra Leonean infants. (DOCX) [file pntd.0011112.s012.docx]

**S10 Table:** **Comparison of study transcript expression levels with those of Sierre Leonean Infants**

| **Transcript** | **Ethiopian Infants** | | **Singh et al (2021)**[1] | |
| --- | --- | --- | --- | --- |
|  | **Mean** | **Median (25^th^, 75^th^ percentiles)** | **Mean** | **Median (25^th^, 75^th^ percentiles)** |
| SI | 2.62 | 0.027 (0.00, 0.087) | - | - |
| Cdx1 | 0.10 | 0.070( 0.027, 0.13) | - | 0.05 (0.03, 0.08) |
| S100A8 | 4.71 | 2.34 (1.15, 5.52) | - | 1.19 (0.53, 2.81) |
| Mucin 12 | 10.68 | 4.48 (2.23, 13.45) | - | - |

**References**

1. Singh A, Potani I, Griswold SP, Suri D, Langlois B, Shen Y, et al. Host fecal mRNAs predicted environmental enteric dysfunction among children with moderate acute malnutrition in Sierra Leone. Am J Trop Med Hyg. 2021;105: 1376–1382. doi:10.4269/ajtmh.21-0348
