# Supplementary material for: Stool biomarkers as measures of enteric pathogen infection in infants from Addis Ababa informal settlements
Source: PLoS Negl Trop Dis. 2023 Feb 21;17(2):e0011112. doi: 10.1371/journal.pntd.0011112 (PMC9983878; doi:10.1371/journal.pntd.0011112)
Supplement: S11 Table — (DOCX) [file pntd.0011112.s013.docx]

**S11 Table:** **Summary statistics of the theory driven scores.**

| **Score** | **Mean +/- SD** | **Min** | **Max** | **Median (25^th^, 75^th^ percentiles)** |
| --- | --- | --- | --- | --- |
| Enterocyte Integrity Score | 5.96 +/- 3.54 | 0 | 12 | 6.00 (3.00, 9.00) |
| Inflammation Score | 7.70 +/-3.19 | 1 | 16 | 8.00 (5.00, 10.00) |
| Acute Score | 5.81 +/- 3.03 | 0 | 12 | 6.00 (3.00, 9.00) |
| Chronic Score | 1.97 +/- 1.42 | 0 | 4 | 2.00 (1.00, 3.00) |
