# Supplementary material for: Stool biomarkers as measures of enteric pathogen infection in infants from Addis Ababa informal settlements
Source: PLoS Negl Trop Dis. 2023 Feb 21;17(2):e0011112. doi: 10.1371/journal.pntd.0011112 (PMC9983878; doi:10.1371/journal.pntd.0011112)
Supplement: S12 Table — (DOCX) [file pntd.0011112.s014.docx]

**S12 Table:** **Summary statistics of the data derived scores.**

| **Score** | **Min** | **Max** | **Median (25^th^, 75^th^ percentiles)** |
| --- | --- | --- | --- |
| Enterocyte Integrity Score | -3.42 | 1.08 | 0.36 (-0.51, 0.71) |
| Chronic Inflammation Score A | -1.37 | 2.62 | -0.13 (-0.79, 0.47) |
| Chronic Inflammation Score B | -1.31 | 2.65 | -0.26 (-0.82, 0.56) |
| Acute Inflammation Score A | -0.81 | 4.47 | -0.34 (-0.62, 0.12) |
| Acute Inflammation Score B | -0.83 | 3.67 | -0.36 (-0.64, 0.22) |
