# Supplementary material for: Stool biomarkers as measures of enteric pathogen infection in infants from Addis Ababa informal settlements
Source: PLoS Negl Trop Dis. 2023 Feb 21;17(2):e0011112. doi: 10.1371/journal.pntd.0011112 (PMC9983878; doi:10.1371/journal.pntd.0011112)
Supplement: S13 Table — (DOCX) [file pntd.0011112.s015.docx]

**S13 Table: Factor loading values and score types for PCA derived using only myeloperoxidase, AAT and neopterin.**

|  | **Protein Biomarker Derived PCA** | |
| --- | --- | --- |
| **Biomarker** | **PC1: Acute Inflammation** | **PC2: Chronic Inflammation** |
| MPO | **0.69** | 0.08 |
| AAT | **0.64** | 0.38 |
| Neopterin | -0.33 | **0.92** |
| Standard deviation | 1.24 | 0.98 |
| Proportion of Variance | 0.51 | 0.32 |
| Cumulative Proportion | 0.51 | 0.83 |
