# Supplementary material for: Stool biomarkers as measures of enteric pathogen infection in infants from Addis Ababa informal settlements
Source: PLoS Negl Trop Dis. 2023 Feb 21;17(2):e0011112. doi: 10.1371/journal.pntd.0011112 (PMC9983878; doi:10.1371/journal.pntd.0011112)
Supplement: S14 Table — (DOCX) [file pntd.0011112.s016.docx]

**S14 Table:** **Number of samples testing positive for each pathogen.**

| **Pathogen** | **6 to 11 months (N=40)** | **12 months and Older (N=96)** | **p-value** |
| --- | --- | --- | --- |
| EAEC_aaiC | 27 | 50 | 0.14 |
| EPEC_eae | 29 | 64 | 0.64 |
| EPEC_bfpA | 28 | 63 | 0.77 |
| ETEC_STh | 6 | 20 | 0.58 |
| STEC_SltII | 4 | 7 | 0.86 |
| *Shigella* | 4 | 22 | 0.13 |
| *Campylobacter* | 5 | 23 | 0.20 |
| ***Giardia*** | **2** | **34** | **<0.001** |
| *Cryptosporidium* | 1 | 9 | 0.30 |
| Noro_GI | 5 | 13 | 0.76 |
| Noro_GII | 18 | 28 | 0.11 |
