# Supplementary material for: Stool biomarkers as measures of enteric pathogen infection in infants from Addis Ababa informal settlements
Source: PLoS Negl Trop Dis. 2023 Feb 21;17(2):e0011112. doi: 10.1371/journal.pntd.0011112 (PMC9983878; doi:10.1371/journal.pntd.0011112)
Supplement: S15 Table — (DOCX) [file pntd.0011112.s017.docx]

**S15 Table: Comparison of Scores by 2-week diarrheal disease prevalence.**

| **Theory Derived Scores** | | | |
| --- | --- | --- | --- |
|  | **Diarrheal** | **Non-Diarrheal** |  |
| **Score** | **Mean (SD)** | **Mean (SD)** | **p-value** |
| Enterocyte Integrity Score | 5.88 (3.49) | 6.01 (3.62) | 0.84 |
| Acute Inflammation Score | 5.87 (3.31) | 5.78 (2.85) | 0.80 |
| Chronic Inflammation Score | 2.11 (1.41) | 1.88 (1.43) | 0.44 |
| Inflammation Score | 8.53 (3.10) | 7.24 (3.16) | 0.08 |
| **Data Derived Scores** | | | |
| Enterocyte Integrity Score | 0.07 (0.94) | -0.03 (1.04) | 0.64 |
| Acute Inflammation Score A | 0.24 (1.20) | -0.12 (0.87) | 0.10 |
| Acute Inflammation Score B | -0.03 (0.94) | 0.01 (1.04) | 0.96 |
| Chronic Inflammation Score A | 0.20 (1.00) | -0.10 (1.00) | 0.16 |
| Chronic Inflammation Score B | 0.14 (0.96) | -0.07 (1.02) | 0.22 |
