# Supplementary material for: Stool biomarkers as measures of enteric pathogen infection in infants from Addis Ababa informal settlements
Source: PLoS Negl Trop Dis. 2023 Feb 21;17(2):e0011112. doi: 10.1371/journal.pntd.0011112 (PMC9983878; doi:10.1371/journal.pntd.0011112)
Supplement: S16 Table — (DOCX) [file pntd.0011112.s018.docx]

| **2-week diarrheal prevalence** | | | |
| --- | --- | --- | --- |
|  | **Non-Diarrheal** | **Diarrheal** | **Prevalence Ratio (95% CI)** |
| EAEC_aaiC | 43 | 34 | 1.10 (0.81 - 1.47) |
| EPEC_eae | 54 | 39 | 1.00 (0.78 - 1.26) |
| EPEC_bfpA | 56 | 35 | 0.87 (0.66 - 1.10) |
| ETEC_Sth | 17 | 9 | 0.51 (0.21 - 1.08) |
| STEC_SltII | 9 | 2 | 0.31 (0.05 - 1.14) |
| *Shigella* | 14 | 12 | 1.19 (0.58 - 2.38) |
| *Campylobacter* | 18 | 10 | 0.73 (0.35 - 1.41) |
| ***Cryptosporidium*** | **2** | **8** | **5.54 ( 1.46 - 35.84)** |
| *Giardia* | 19 | 17 | 1.24 (0.70 - 2.18) |
| Noro_GI | 12 | 6 | 0.69 (0.25 - 1.67) |
| Noro_GII | 26 | 20 | 1.07 (0.65 - 1.71) |

**S16 Table: Unadjusted associations between pathogen prevalence and 2-week diarrheal disease prevalence. Significant associations are bolded.**
