# Supplementary material for: Stool biomarkers as measures of enteric pathogen infection in infants from Addis Ababa informal settlements
Source: PLoS Negl Trop Dis. 2023 Feb 21;17(2):e0011112. doi: 10.1371/journal.pntd.0011112 (PMC9983878; doi:10.1371/journal.pntd.0011112)
Supplement: S1 Fig — (PDF) [file pntd.0011112.s019.pdf]

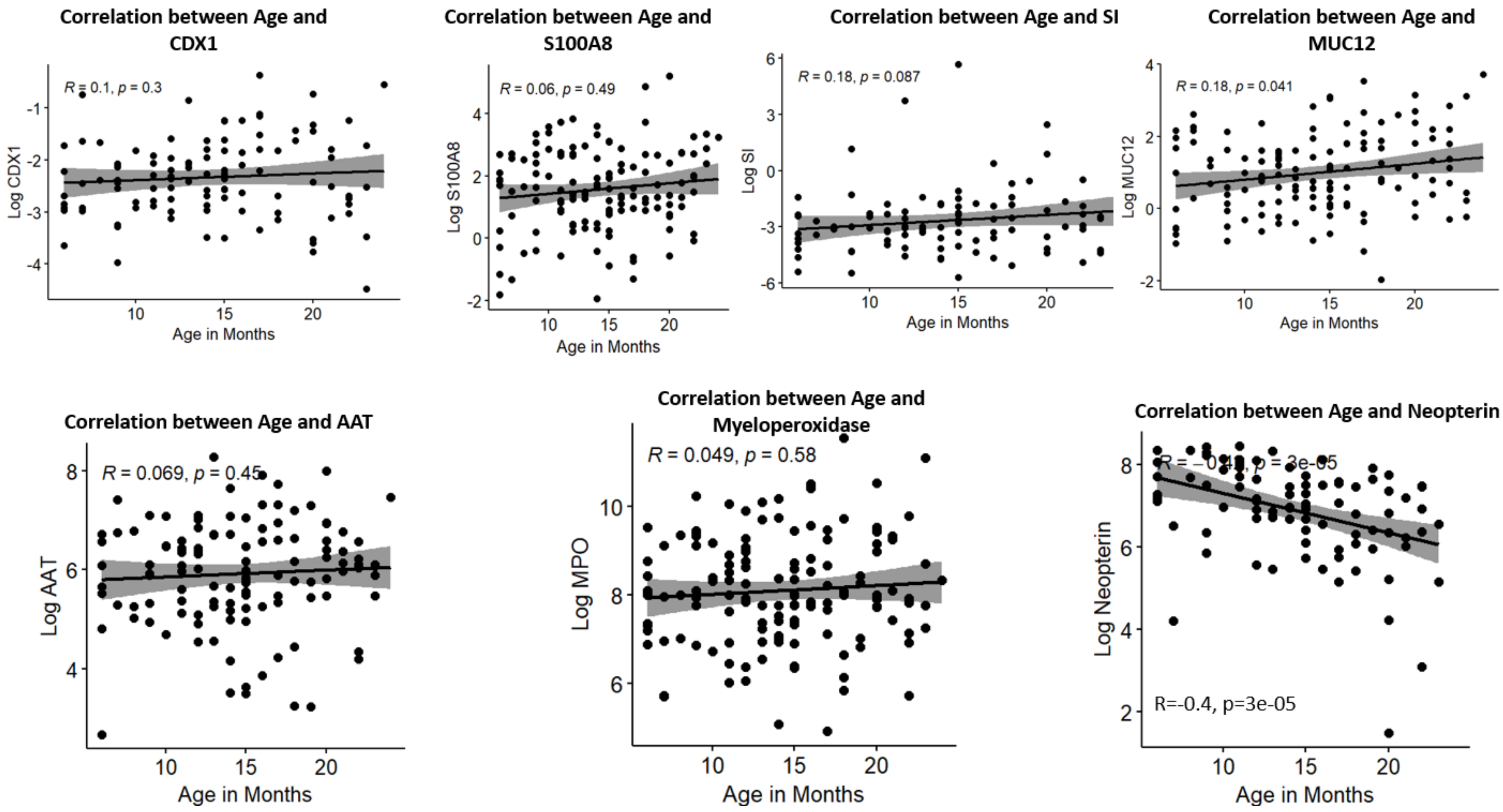

Figure S1: Correlation between age and biomarker levels in the full sample. Only neopterin and MUC12 showed significant correlations with age.
