# Supplementary material for: Stool biomarkers as measures of enteric pathogen infection in infants from Addis Ababa informal settlements
Source: PLoS Negl Trop Dis. 2023 Feb 21;17(2):e0011112. doi: 10.1371/journal.pntd.0011112 (PMC9983878; doi:10.1371/journal.pntd.0011112)
Supplement: S2 Fig — (PDF) [file pntd.0011112.s020.pdf]

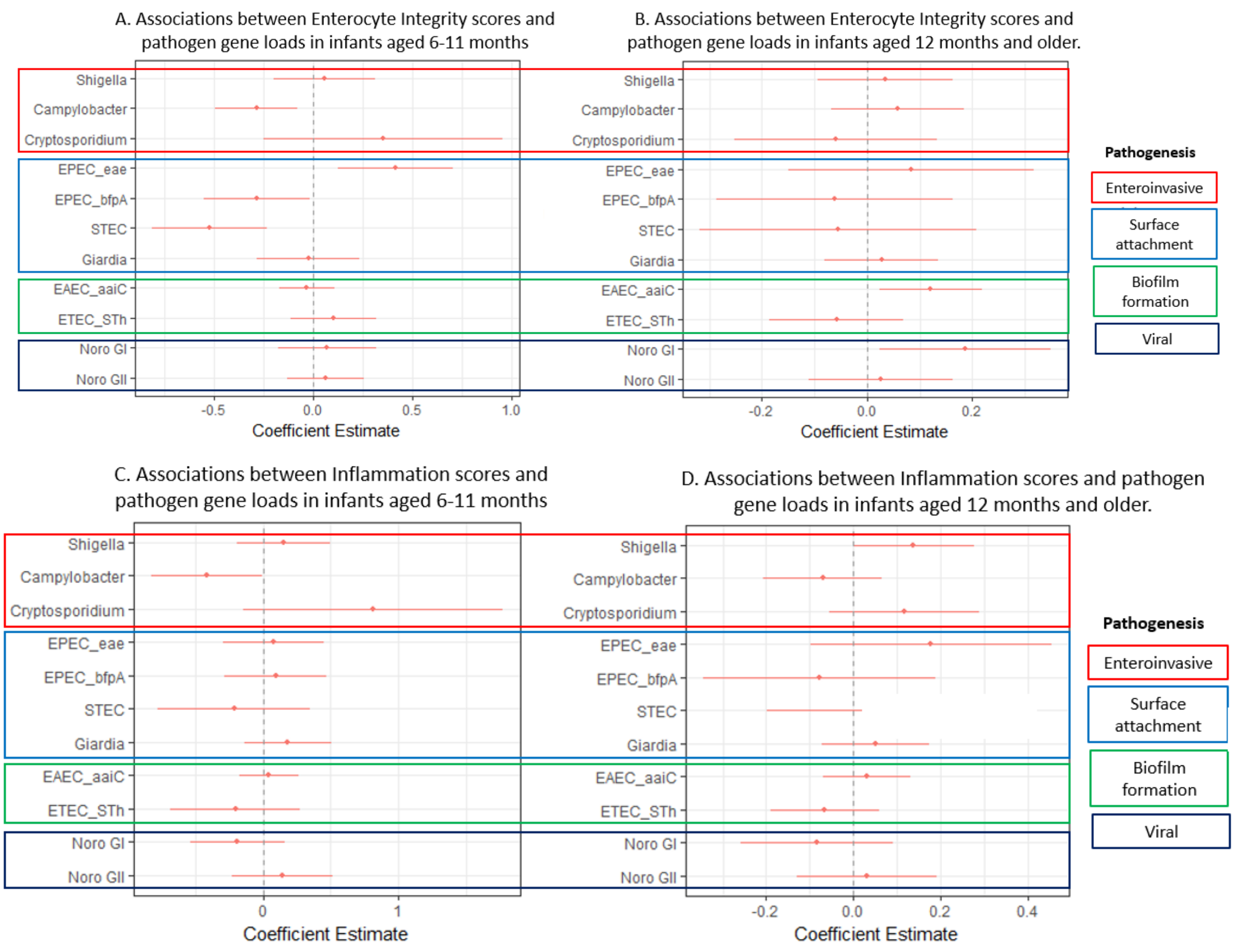

A. Associations between Enterocyte Integrity scores and pathogen gene loads in infants aged 6-11 months
B. Associations between Enterocyte Integrity scores and pathogen gene loads in infants aged 12 months and older.
C. Associations between Inflammation scores and pathogen gene loads in infants aged 6-11 months
D. Associations between Inflammation scores and pathogen gene loads in infants aged 12 months and older.
Shigella
Campylobacter
Cryptosporidium
EPEC_eae
EPEC_bfpA
STEC
Giardia
EAEC_aaiC
ETEC_STh
Noro GI
Noro GII
Coefficient Estimate
Pathogenesis
Enteroinvasive
Surface attachment
Biofilm formation
Viral

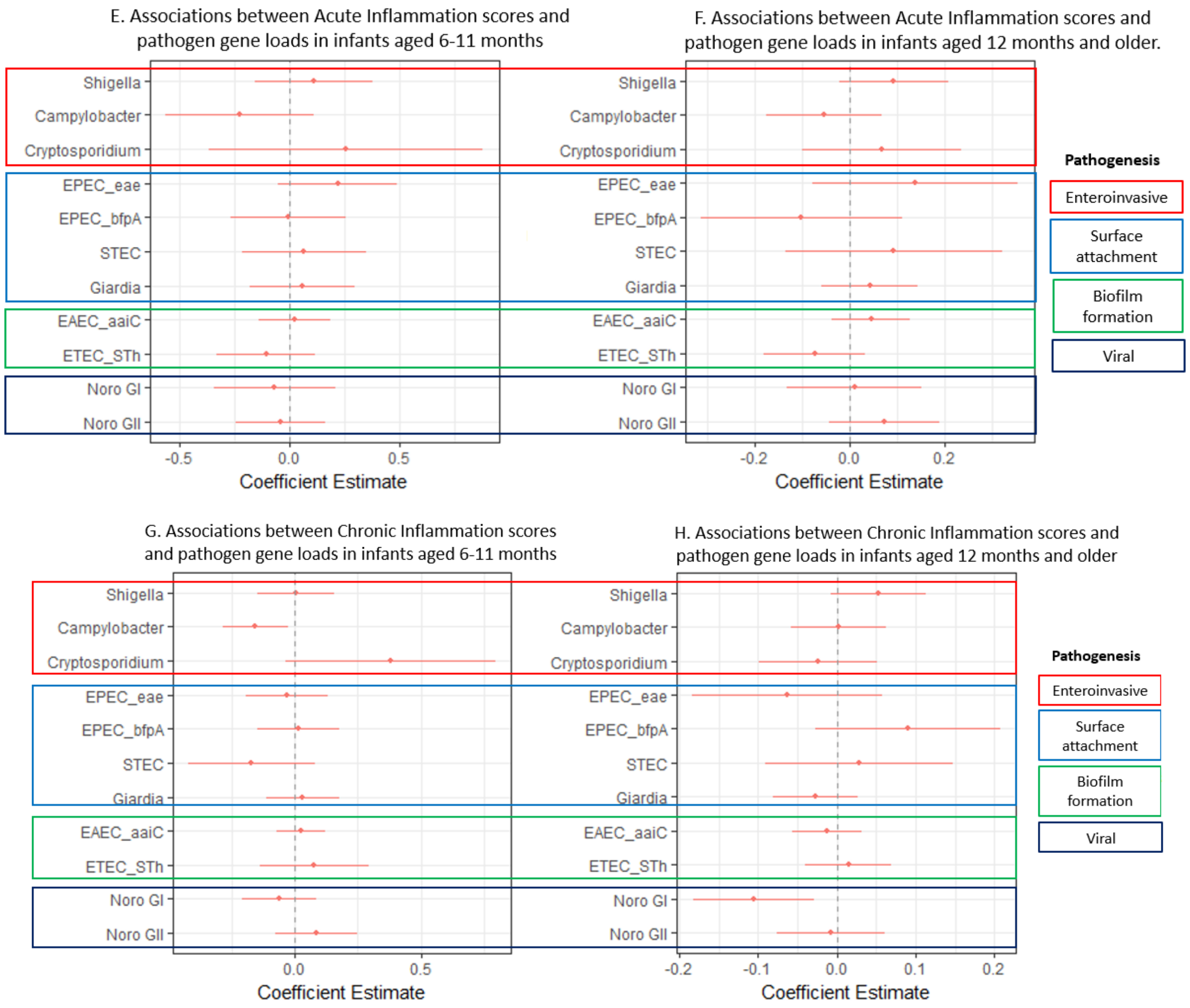

S2 Fig: Associations between the theory driven score and age stratified stool pathogen gene counts; (a-b) associations between stool pathogen gene counts and the Enterocyte Integrity Score, (c-d) associations between the overall Inflammation Score and stool pathogen gene counts, (e-f) the associations between stool pathogen gene counts and Acute Inflammation, and (g-h) associations between the Chronic Inflammation Score and stool pathogen counts.
