# Supplementary material for: Stool biomarkers as measures of enteric pathogen infection in infants from Addis Ababa informal settlements
Source: PLoS Negl Trop Dis. 2023 Feb 21;17(2):e0011112. doi: 10.1371/journal.pntd.0011112 (PMC9983878; doi:10.1371/journal.pntd.0011112)
Supplement: S3 Fig — (PDF) [file pntd.0011112.s021.pdf]

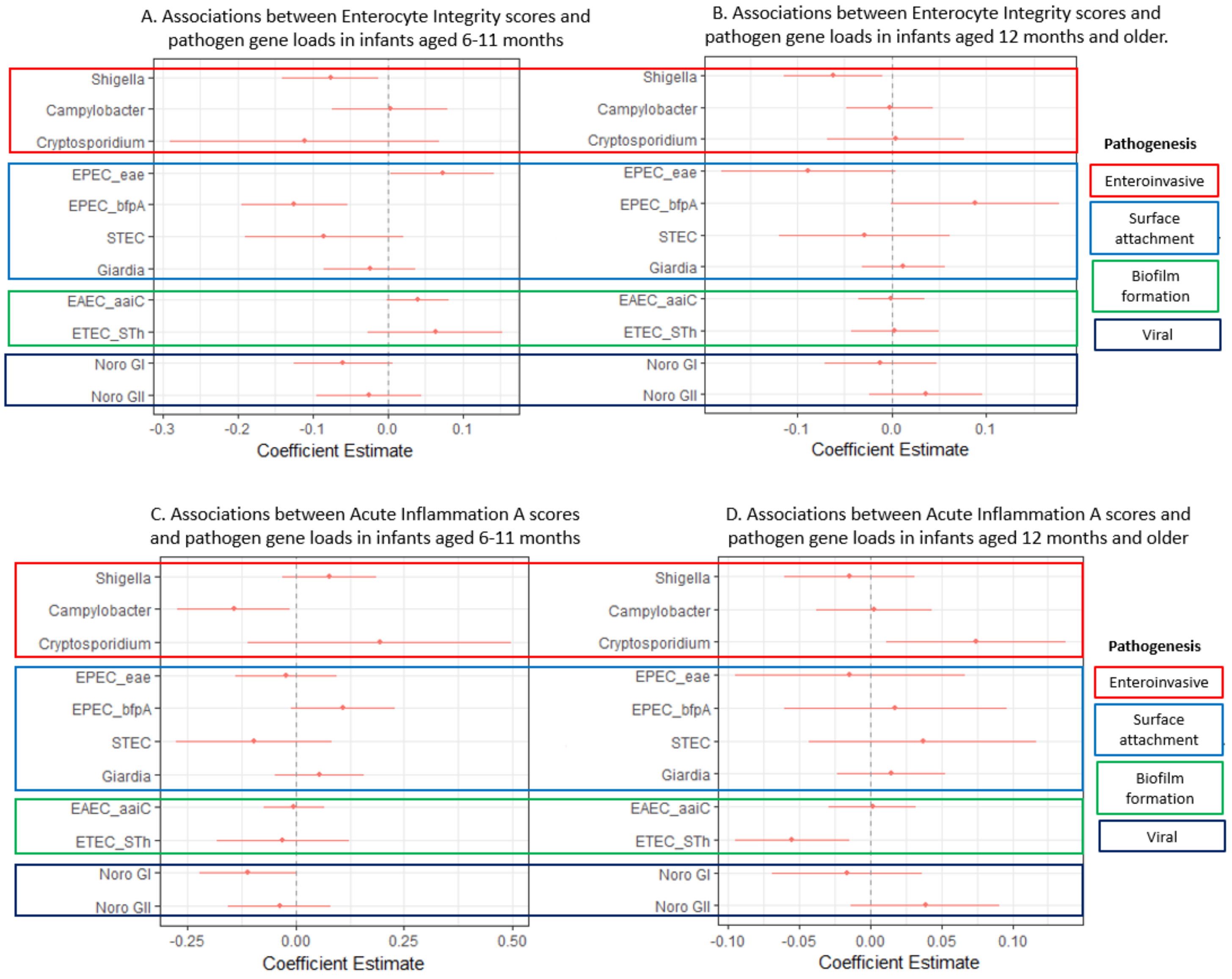
A. Associations between Enterocyte Integrity scores and pathogen gene loads in infants aged 6-11 months
B. Associations between Enterocyte Integrity scores and pathogen gene loads in infants aged 12 months and older.
C. Associations between Acute Inflammation A scores and pathogen gene loads in infants aged 6-11 months
D. Associations between Acute Inflammation A scores and pathogen gene loads in infants aged 12 months and older
Shigella
Campylobacter
Cryptosporidium
EPEC_eae
EPEC_bfpA
STEC
Giardia
EAEC_aaiC
ETEC_STh
Noro GI
Noro GII
-0.3
-0.2
-0.1
0.0
0.1
-0.25
0.00
0.25
0.50
-0.10
-0.05
0.00
0.05
0.10
Coefficient Estimate
Pathogenesis
Enteroinvasive
Surface attachment
Biofilm formation
Viral

E. Associations between Acute Inflammation B scores and pathogen gene loads in infants aged 6-11 months.

F. Associations between Acute Inflammation B scores and pathogen gene loads in infants aged 12 months and older.

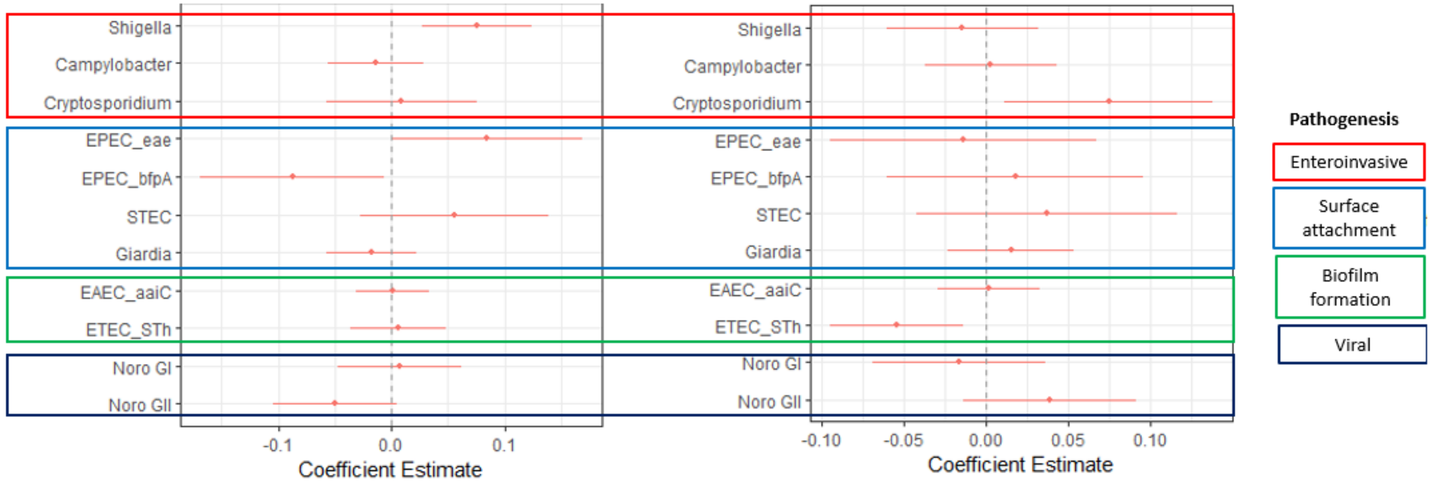


G. Associations between Chronic Inflammation A scores and pathogen gene loads in infants aged 6-11 months.

H. Associations between Chronic Inflammation A scores and pathogen gene loads in infants aged 12 months and older.

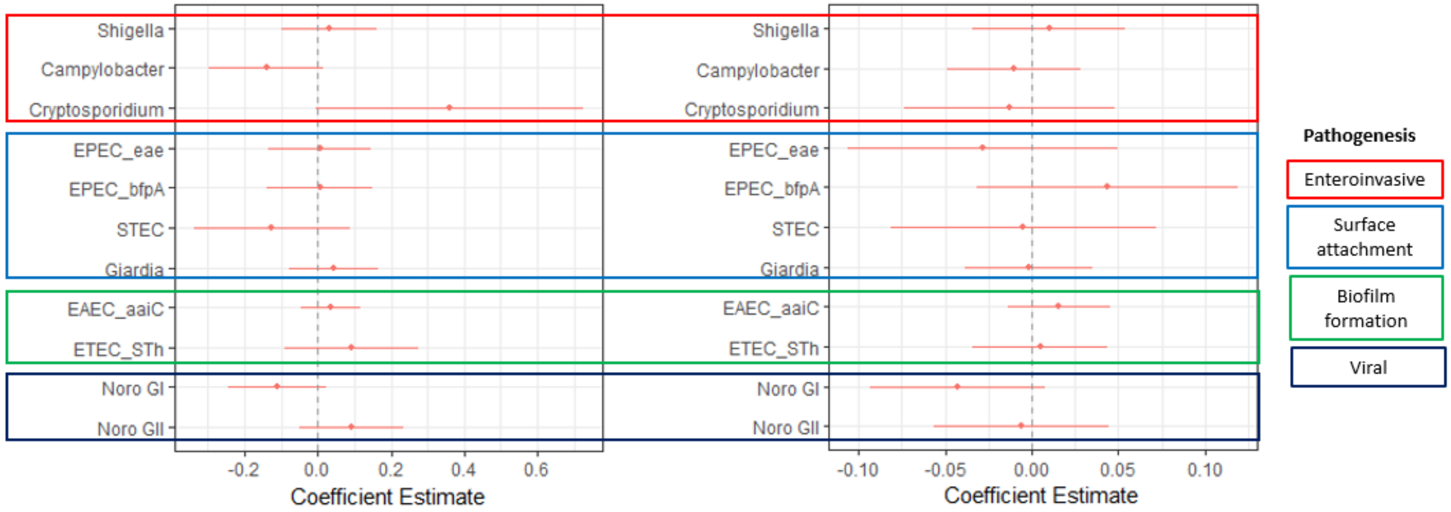

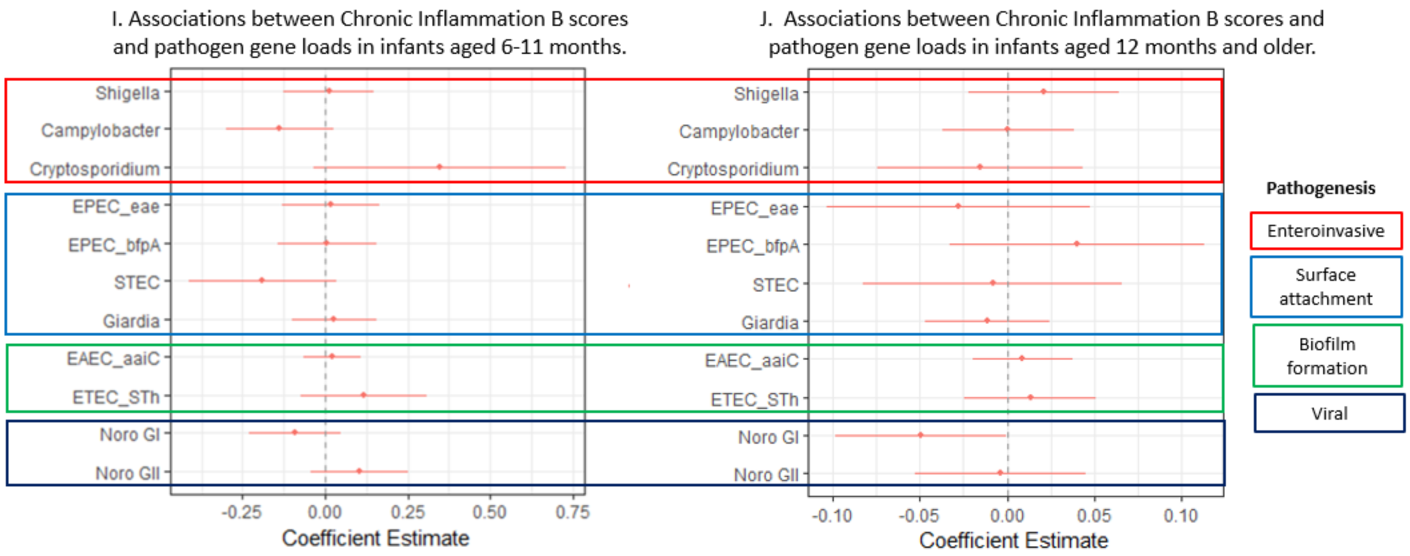


**S3 Fig: Associations between the data derived score and age stratified stool pathogen gene counts; (a-b) associations between Enterocyte Integrity Score and stool pathogen gene counts, (c-d) associations between Acute Inflammation Score A and stool pathogen gene counts (e-f) associations between Acute Inflammation Score B and stool pathogen gene counts, (g-h) associations between stool pathogen gene counts and the Chronic Inflammation Score A , and (i-j) associations between stool pathogen gene counts and Chronic Inflammation Score B.**
