# Supplementary material for: Stool biomarkers as measures of enteric pathogen infection in infants from Addis Ababa informal settlements
Source: PLoS Negl Trop Dis. 2023 Feb 21;17(2):e0011112. doi: 10.1371/journal.pntd.0011112 (PMC9983878; doi:10.1371/journal.pntd.0011112)
Supplement: S4 Fig — (PDF) [file pntd.0011112.s022.pdf]

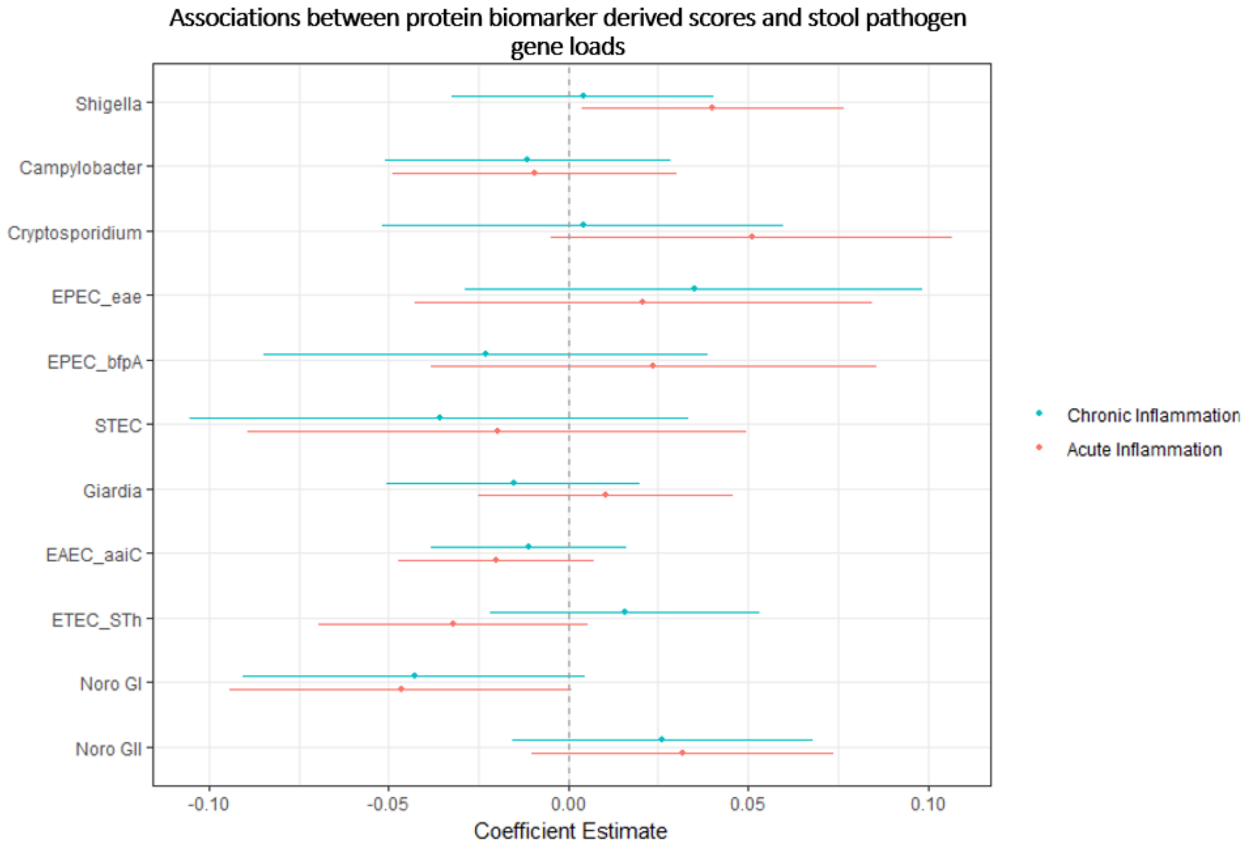

S4 Fig: Associations between the protein biomarker-based data derived score and stool pathogen gene counts. Only *Shigella* gene counts were significantly associated with the scores.
