# Supplementary material for: Stool biomarkers as measures of enteric pathogen infection in infants from Addis Ababa informal settlements
Source: PLoS Negl Trop Dis. 2023 Feb 21;17(2):e0011112. doi: 10.1371/journal.pntd.0011112 (PMC9983878; doi:10.1371/journal.pntd.0011112)
Supplement: S5 Fig — (PDF) [file pntd.0011112.s023.pdf]

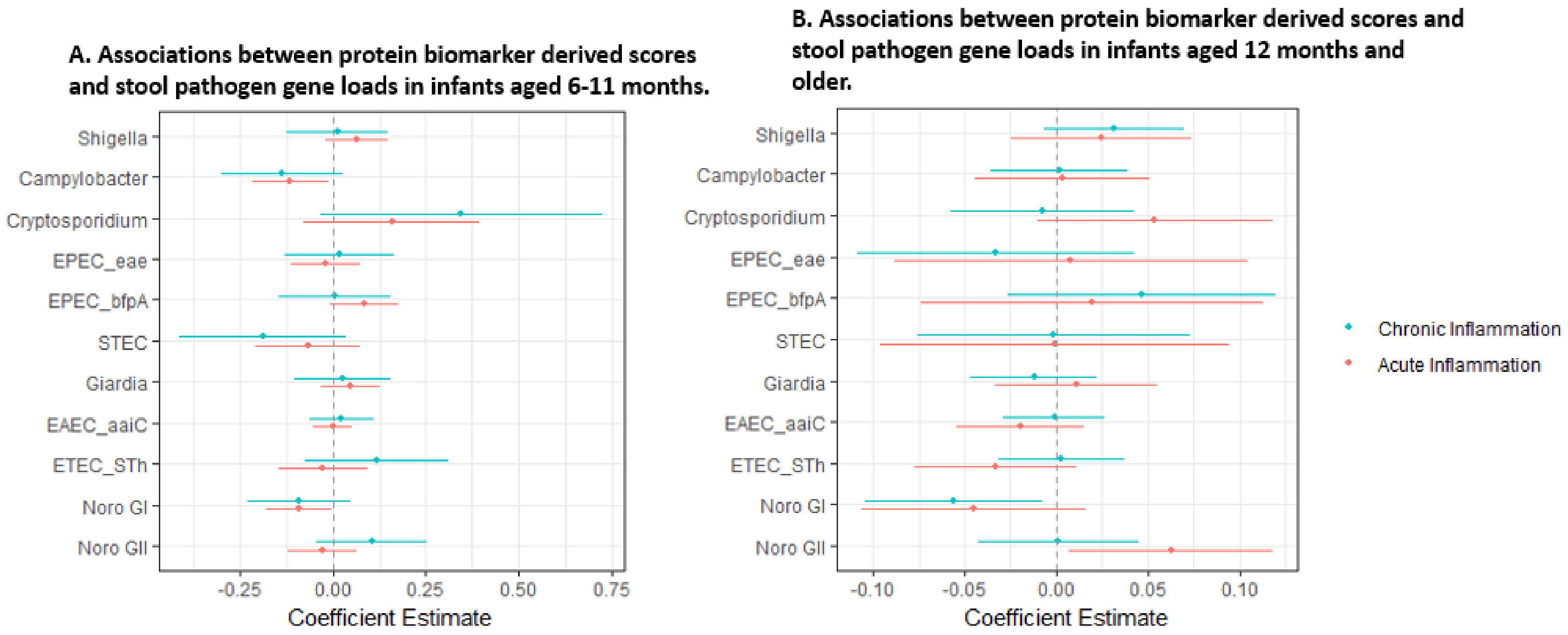


**S5 Fig: Associations between the protein biomarker-based data derived score and stool pathogen gene counts in a) infants aged 6-11 months and b) in infants aged 12 months and older.**
